# Delayed Mucosal Antiviral Responses Despite Robust Peripheral Inflammation in Fatal COVID-19

**DOI:** 10.1093/infdis/jiad590

**Published:** 2023-12-22

**Authors:** Jasmin K Sidhu, Matthew K Siggins, Felicity Liew, Clark D Russell, Ashley S S Uruchurtu, Christopher Davis, Lance Turtle, Shona C Moore, Hayley E Hardwick, Wilna Oosthuyzen, Emma C Thomson, Malcolm G Semple, J Kenneth Baillie, Peter J M Openshaw, Ryan S Thwaites, ISARIC4C investigators, J Kenneth Baillie, Peter J M Openshaw, Malcolm G Semple, Beatrice Alex, Petros Andrikopoulos, Benjamin Bach, Wendy S Barclay, Debby Bogaert, Meera Chand, Kanta Chechi, Graham S Cooke, Ana da Silva Filipe, Thushan de Silva, Annemarie B Docherty, Gonçalo dos Santos Correia, Marc-Emmanuel Dumas, Jake Dunning, Tom Fletcher, Christoper A Green, William Greenhalf, Julian L Griffin, Rishi K Gupta, Ewen M Harrison, Antonia Ying Wai Ho, Karl Holden, Peter W Horby, Samreen Ijaz, Saye Khoo, Paul Klenerman, Andrew Law, Matthew R Lewis, Sonia Liggi, Wei Shen Lim, Lynn Maslen, Alexander J Mentzer, Laura Merson, Alison M Meynert, Shona C Moore, Mahdad Noursadeghi, Michael Olanipekun, Anthonia Osagie, Massimo Palmarini, Carlo Palmieri, William A Paxton, Georgios Pollakis, Nicholas Price, Andrew Rambaut, David L Robertson, Clark D Russell, Vanessa Sancho-Shimizu, Caroline J Sands, Janet T Scott, Louise Sigfrid, Tom Solomon, Shiranee Sriskandan, David Stuart, Charlotte Summers, Olivia V Swann, Zoltan Takats, Panteleimon Takis, Richard S Tedder, A A Roger Thompson, Emma C Thomson, Ryan S Thwaites, Lance C W Turtle, Maria Zambon, Thomas M Drake, Cameron J Fairfield, Stephen R Knight, Kenneth A Mclean, Derek Murphy, Lisa Norman, Riinu Pius, Catherine A Shaw, Marie Connor, Jo Dalton, Carrol Gamble, Michelle Girvan, Sophie Halpin, Janet Harrison, Clare Jackson, James Lee, Laura Marsh, Daniel Plotkin, Stephanie Roberts, Egle Saviciute, Sara Clohisey, Ross Hendry, Susan Knight, Eva Lahnsteiner, Andrew Law, Gary Leeming, Lucy Norris, James Scott-Brown, Sarah Tait, Murray Wham, Gail Carson, Richard Clark, Audrey Coutts, Lorna Donnelly, Angie Fawkes, Tammy Gilchrist, Katarzyna Hafezi, Louise MacGillivray, Alan Maclean, Sarah McCafferty, Kirstie Morrice, Lee Murphy, Nicola Wrobel, Kayode Adeniji, Daniel Agranoff, Ken Agwuh, Dhiraj Ail, Erin L Aldera, Ana Alegria, Sam Allen, Brian Angus, Abdul Ashish, Dougal Atkinson, Shahedal Bari, Gavin Barlow, Stella Barnass, Nicholas Barrett, Christopher Bassford, Sneha Basude, David Baxter, Michael Beadsworth, Jolanta Bernatoniene, John Berridge, Colin Berry, Nicola Best, Pieter Bothma, Robin Brittain-Long, Naomi Bulteel, Tom Burden, Andrew Burtenshaw, Vikki Caruth, David Chadwick, David Chadwick, Duncan Chambler, Nigel Chee, Jenny Child, Srikanth Chukkambotla, Tom Clark, Paul Collini, Catherine Cosgrove, Jason Cupitt, Maria-Teresa Cutino-Moguel, Paul Dark, Chris Dawson, Samir Dervisevic, Phil Donnison, Sam Douthwaite, Andrew Drummond, Ingrid DuRand, Ahilanadan Dushianthan, Tristan Dyer, Cariad Evans, Chi Eziefula, Chrisopher Fegan, Adam Finn, Duncan Fullerton, Sanjeev Garg, Atul Garg, Effrossyni Gkrania-Klotsas, Jo Godden, Arthur Goldsmith, Clive Graham, Tassos Grammatikopoulos, Elaine Hardy, Stuart Hartshorn, Daniel Harvey, Peter Havalda, Daniel B Hawcutt, Maria Hobrok, Luke Hodgson, Anil Hormis, Joanne Howard, Michael Jacobs, Susan Jain, Paul Jennings, Agilan Kaliappan, Vidya Kasipandian, Stephen Kegg, Michael Kelsey, Jason Kendall, Caroline Kerrison, Ian Kerslake, Oliver Koch, Gouri Koduri, George Koshy, Shondipon Laha, Steven Laird, Susan Larkin, Tamas Leiner, Patrick Lillie, James Limb, Vanessa Linnett, Jeff Little, Mark Lyttle, Michael MacMahon, Emily MacNaughton, Ravish Mankregod, Huw Masson, Elijah Matovu, Katherine McCullough, Ruth McEwen, Manjula Meda, Gary Mills, Jane Minton, Kavya Mohandas, Quen Mok, James Moon, Elinoor Moore, Patrick Morgan, Craig Morris, Katherine Mortimore, Samuel Moses, Mbiye Mpenge, Rohinton Mulla, Michael Murphy, Thapas Nagarajan, Megan Nagel, Mark Nelson, Lillian Norris, Matthew K O'Shea, Marlies Ostermann, Igor Otahal, Mark Pais, Carlo Palmieri, Selva Panchatsharam, Danai Papakonstantinou, Padmasayee Papineni, Hassan Paraiso, Brij Patel, Natalie Pattison, Justin Pepperell, Mark Peters, Mandeep Phull, Stefania Pintus, Tim Planche, Frank Post, David Price, Rachel Prout, Nikolas Rae, Henrik Reschreiter, Tim Reynolds, Neil Richardson, Mark Roberts, Devender Roberts, Alistair Rose, Guy Rousseau, Bobby Ruge, Brendan Ryan, Taranprit Saluja, Sarah Cole, Matthias L Schmid, Aarti Shah, Manu Shankar-Hari, Prad Shanmuga, Anil Sharma, Anna Shawcross, Jagtur Singh Pooni, Jeremy Sizer, Richard Smith, Catherine Snelson, Nick Spittle, Nikki Staines, Tom Stambach, Richard Stewart, Pradeep Subudhi, Tamas Szakmany, Kate Tatham, Jo Thomas, Chris Thompson, Robert Thompson, Ascanio Tridente, Darell Tupper-Carey, Mary Twagira, Nick Vallotton, Rama Vancheeswaran, Rachel Vincent, Lisa Vincent-Smith, Shico Visuvanathan, Alan Vuylsteke, Sam Waddy, Rachel Wake, Andrew Walden, Ingeborg Welters, Tony Whitehouse, Paul Whittaker, Ashley Whittington, Meme Wijesinghe, Martin Williams, Lawrence Wilson, Stephen Winchester, Martin Wiselka, Adam Wolverson, Daniel G Wootton, Andrew Workman, Bryan Yates, Peter Young, Sarah E McDonald, Victoria Shaw, Katie A Ahmed, Jane A Armstrong, Milton Ashworth, Innocent G Asiimwe, Siddharth Bakshi, Samantha L Barlow, Laura Booth, Benjamin Brennan, Katie Bullock, Nicola Carlucci, Emily Cass, Benjamin WA Catterall, Jordan J Clark, Emily A Clarke, Sarah Cole, Louise Cooper, Helen Cox, Christopher Davis, Oslem Dincarslan, Alejandra Doce Carracedo, Chris Dunn, Philip Dyer, Angela Elliott, Anthony Evans, Lorna Finch, Lewis W S Fisher, Lisa Flaherty, Terry Foster, Isabel Garcia-Dorival, Philip Gunning, Catherine Hartley, Anthony Holmes, Rebecca L Jensen, Christopher B Jones, Trevor R Jones, Shadia Khandaker, Katharine King, Robyn T Kiy, Chrysa Koukorava, Annette Lake, Suzannah Lant, Diane Latawiec, Lara Lavelle-Langham, Daniella Lefteri, Lauren Lett, Lucia A Livoti, Maria Mancini, Hannah Massey, Nicole Maziere, Sarah McDonald, Laurence McEvoy, John McLauchlan, Soeren Metelmann, Nahida S Miah, Joanna Middleton, Joyce Mitchell, Shona C Moore, Ellen G Murphy, Rebekah Penrice-Randal, Jack Pilgrim, Tessa Prince, Will Reynolds, P Matthew Ridley, Debby Sales, Victoria E Shaw, Rebecca K Shears, Benjamin Small, Krishanthi S Subramaniam, Agnieska Szemiel, Aislynn Taggart, Jolanta Tanianis-Hughes, Jordan Thomas, Erwan Trochu, Libby van Tonder, Eve Wilcock, J Eunice Zhang, Seán Keating, Cara Donegan, Rebecca G Spencer, Primrose Chikowore, Chloe Donohue, Fiona Griffiths, Hayley Hardwick, Wilna Oosthuyzen

**Affiliations:** National Heart and Lung Institute, Imperial College London, London, United Kingdom; National Heart and Lung Institute, Imperial College London, London, United Kingdom; National Heart and Lung Institute, Imperial College London, London, United Kingdom; Centre for Inflammation Research, University of Edinburgh, Edinburgh, United Kingdom; National Heart and Lung Institute, Imperial College London, London, United Kingdom; Medical Research Council Centre for Virus Research, University of Glasgow, Glasgow, United Kingdom; Department of Clinical Infection, Microbiology, and Immunology, University of Liverpool, Liverpool, United Kingdom; Tropical and Infectious Disease Unit, Liverpool University Hospitals NHS Foundation Trust, Liverpool Health Partners, Liverpool, United Kingdom; Department of Clinical Infection, Microbiology, and Immunology, University of Liverpool, Liverpool, United Kingdom; Department of Clinical Infection, Microbiology, and Immunology, University of Liverpool, Liverpool, United Kingdom; Roslin Institute, University of Edinburgh, Edinburgh, United Kingdom; Medical Research Council Centre for Virus Research, University of Glasgow, Glasgow, United Kingdom; Department of Clinical Research, London School of Hygiene and Tropical Medicine, London, United Kingdom; National Institute for Health and Care Research Health Protection Research Unit in Emerging and Zoonotic Infections, Institute of Infection, Veterinary, and Ecological Sciences, Faculty of Health and Life Sciences, University of Liverpool, Liverpool, United Kingdom; Respiratory Medicine, Alder Hey Children's Hospital, Liverpool, United Kingdom; Roslin Institute, University of Edinburgh, Edinburgh, United Kingdom; Intensive Care Unit, Royal Infirmary Edinburgh, Edinburgh, United Kingdom; National Heart and Lung Institute, Imperial College London, London, United Kingdom; National Heart and Lung Institute, Imperial College London, London, United Kingdom

**Keywords:** COVID-19, SARS-CoV-2, virus, cytokine, chemokine, mucosa, airway, nose, lung

## Abstract

**Background:**

While inflammatory and immune responses to severe acute respiratory syndrome coronavirus 2 (SARS-CoV-2) infection in peripheral blood are extensively described, responses at the upper respiratory mucosal site of initial infection are relatively poorly defined. We sought to identify mucosal cytokine/chemokine signatures that distinguished coronavirus disease 2019 (COVID-19) severity categories, and relate these to disease progression and peripheral inflammation.

**Methods:**

We measured 35 cytokines and chemokines in nasal samples from 274 patients hospitalized with COVID-19. Analysis considered the timing of sampling during disease, as either the early (0–5 days after symptom onset) or late (6–20 days after symptom onset) phase.

**Results:**

Patients that survived severe COVID-19 showed interferon (IFN)-dominated mucosal immune responses (IFN-γ, CXCL10, and CXCL13) early in infection. These early mucosal responses were absent in patients who would progress to fatal disease despite equivalent SARS-CoV-2 viral load. Mucosal inflammation in later disease was dominated by interleukin 2 (IL-2), IL-10, IFN-γ, and IL-12p70, which scaled with severity but did not differentiate patients who would survive or succumb to disease. Cytokines and chemokines in the mucosa showed distinctions from responses evident in the peripheral blood, particularly during fatal disease.

**Conclusions:**

Defective early mucosal antiviral responses anticipate fatal COVID-19 but are not associated with viral load. Early mucosal immune responses may define the trajectory of severe COVID-19.

## Teaser

Fatal COVID-19 is associated robust peripheral inflammation; however, early interferon-led responses in the respiratory mucosa are delayed relative to those that will survive severe SARS-CoV-2 infection.

## Graphical Abstract

**Graphical Abstract:**
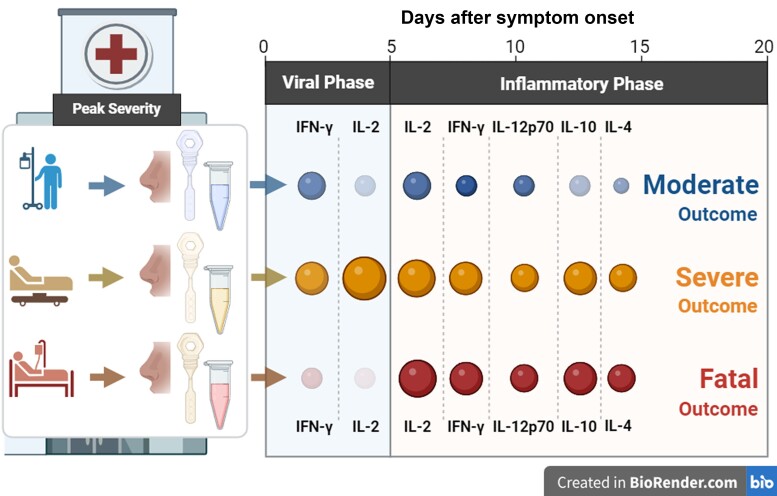

Immunopathogenesis is a dominant feature of severe coronavirus disease 2019 (COVID-19) and hospitalized patients with established disease benefit from steroidal and anti-inflammatory therapies. In late-stage disease, immunopathogenesis is increasingly well characterized but less is known about early mucosal immune responses. In respiratory viral infections the upper respiratory tract (URT) is considered the site of initial viral replication, with preexisting adaptive immunity preventing or limiting infection [1]. Interferons (IFNs) and innate immune effector cells provide the next layer of protection and are followed by adaptive T- and B-cell responses [2–4]. Severe acute respiratory syndrome coronavirus 2 (SARS-CoV-2) infection has been associated with an inadequate, delayed, or prolonged innate response, which can in turn delay adaptive responses [4, 5].

The value of immunomodulation in COVID-19 was first observed as reduced 28-day mortality following administration of dexamethasone to patients receiving oxygen or supportive ventilation [6]. Inhibition of interleukin 6 (IL-6) with monoclonal antibodies further demonstrated the benefit of immunosuppressive therapies in COVID-19 [7]. An alternative to immunosuppression of established disease is the enhancement of protective immune responses, and interventions prior to or during early hospitalization are desirable. Disruption of viral replication has been studied for this aim; however, the antiviral molnupiravir performs no better than placebo at preventing hospitalizations within at-risk groups [8]. Symptomatic nonhospitalized SARS-CoV-2 patients treated <5 days after symptom onset with the anti–SARS-CoV-2 monoclonal sotrovimab demonstrated a reduction in subsequent hospitalizations [9], supporting the principal that early intervention may reduce severity. However, antivirals and monoclonals risk decreasing efficacy due to viral escape mutations. Additionally, whilst higher viral load might be reasonably thought to influence severity, the relationship between URT viral load and severity is unclear [10–12]. Boosting host antiviral responses with pegylated IFN-λ within 7 days of symptom onset was effective in older participants at higher risk of severe disease [13]. While protective and harmful immune responses during COVID-19 are well described in blood, human mucosal immune responses during SARS-CoV-2 infection are relatively understudied. This has limited the development of immunomodulatory therapies that could enhance protective mucosal responses, inhibit immunopathogenesis, or define early prognostic markers.

The interval between SARS-CoV-2 exposure and symptom onset has been estimated at 5 days, although this shortened as viral variants adapted to human transmission [14]. Therefore, the symptomatic period has been considered to transition from a virus-initiated early viral phase (<5 days of symptoms) to an inflammatory phase [15]. The nasal mucosa offers a uniquely accessible window into the respiratory tract and, unlike lower airway sampling, can be performed repeatedly in large cohorts, enabling the characterization of rare disease outcomes. We used nasosorption sampling in a large prospective multicenter cohort hospitalized with COVID-19 to investigate the immune response in the upper airway and compared these responses to peripheral blood [16]. We now describe the temporal pattern of responses in participants of diverse severity, highlighting initial transient mucosal hyporesponsiveness in participants that progress to fatal disease.

## METHODS

### Study Design

The International Severe Acute Respiratory Infection Consortium (ISARIC) World Health Organization (WHO) Clinical Characterization Protocol for Severe Emerging Infections in the UK (CCP-UK) is a prospective cohort study of patients hospitalized with COVID-19, with recruitment across 258 hospitals in England, Scotland, and Wales (National Institute for Health Research Clinical Research Network Central Portfolio Management System ID, 14152) [17]. The protocol, revision history, case report form, patient information leaflets, consent forms, and details of the Independent Data and Material Access Committee are available online [18]. This was a prepositioned pandemic preparedness study with urgent public health research status [17]. Additional detail on the study design and analyses can be found in the Supplementary Materials.

## RESULTS

### Elevated Mucosal Proinflammatory Cytokine Levels in Severe and Fatal COVID-19

Prepandemic healthy controls (HCs, n = 25) and patients hospitalized with COVID-19 within 20 days of symptom onset (n = 274) provided nasal mucosal samples (Supplementary Table 1). Patients were categorized into 3 groups based upon peak severity using the WHO clinical progression scale [19]: moderate disease (n = 142) encompassing no oxygen therapy (WHO score 4) and oxygenation by mask or nasal prongs (WHO score 5); severe disease (n = 92) including noninvasive ventilation or high-flow nasal cannula oxygenation (WHO score 6) and invasive mechanical ventilation (WHO scores 7–9); and fatal disease (WHO score 10; n = 40). In agreement with the wider ISARIC4C cohort [20], fatal outcome was associated with older age and male sex (Supplementary Table 1).

**Figure 1.**
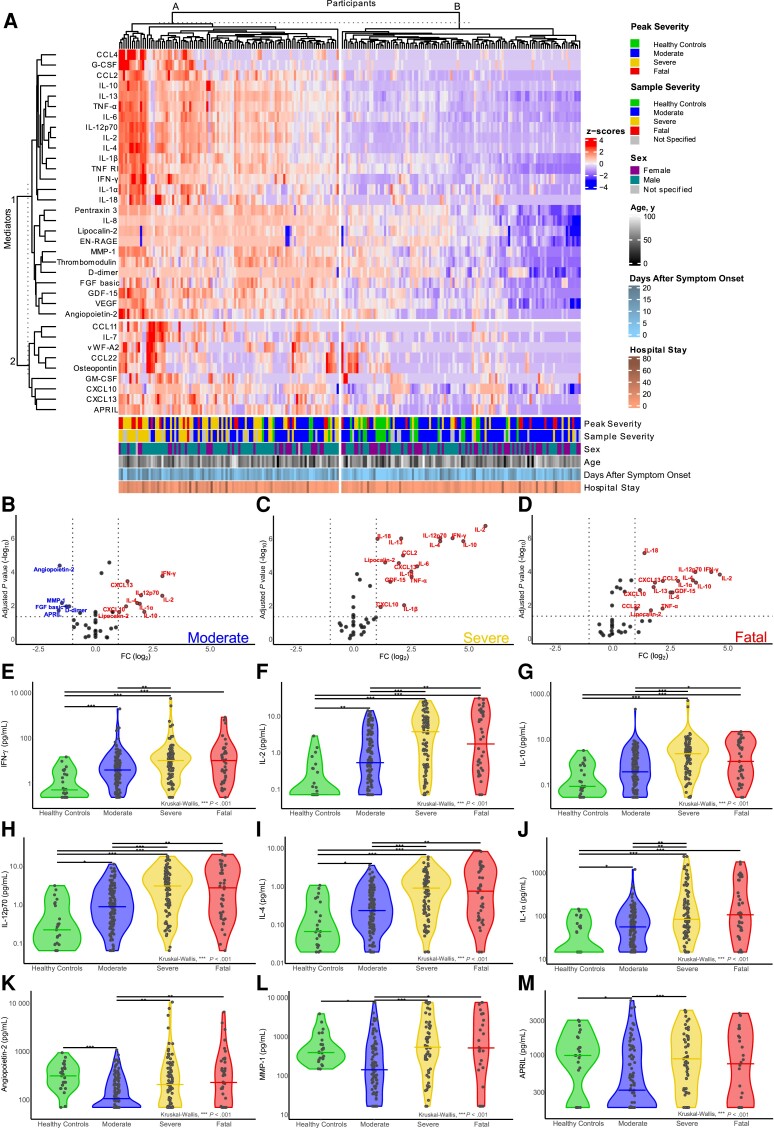
COVID-19 severity was associated with clusters of elevated inflammatory markers in the nasal mucosa. *A*, K-means clustered heatmap of 35 cytokines and chemokines in the nasal mucosa of healthy controls (n = 25) and patients hospitalized with COVID-19 (n = 215). Annotations provide participant peak severity (peak severity), severity at the time of sample collection (sample severity), sex, age, duration of symptoms at the time of sample collection (days after symptom onset), and total duration of hospital stay (hospital stay) in days. *B*–*D*, Nasal cytokine and chemokine levels in patients hospitalized with COVID-19 (n = 274) shown as volcano plots relative to healthy controls for (*B*) moderate, (*C*) severe, and (*D*) fatal peak COVID-19 severity groups. Data shown are −log_10_ transformed false discovery rate adjusted Wilcoxon rank-sum *P* values (horizontal line at cutoff ≥.05) plotted against log_2_ fold changes (vertical lines at cutoffs ≤ −2 and ≥2) for nasal samples taken at 0–20 days after symptom onset. *E*–*M*, Individual cytokines and chemokines of particular interest in each COVID-19 peak-severity group and healthy controls: (*E*) IFN-γ, (*F*) IL-2, (*G*) IL-10, (*H*) IL-12p70, (*I*) IL-4, (*J*) IL-1α, (*K*) angiopoietin-2, (*L*) MMP-1, and (*M*) APRIL. Group median levels are shown as lines and significance levels determined using Kruskal-Wallis tests with Dunn's *P* value correction. *B*–*M*, Data from the first time point per participant are shown. *P < .05, **P < .01, ***P < .001. Abbreviations: APRIL, A proliferation-inducing ligand; COVID-19, coronavirus disease 2019; FC, fold change; FGF, fibroblast growth factor; G-CSF, granulocyte-colony-stimulating factor; GDF-15, growth differentiation factor 15; GM-CSF, granulocyte-macrophage colony-stimulating factor; IL, interleukin; IFN-γ, interferon-γ; MMP-1, matrix metalloproteinase-1; TNF-α, tumor necrosis factor-α; VEGF, vascular endothelial growth factor.

To identify associations between severity and nasal cytokines/chemokines, K-means clustering and principal component analysis (PCA) were performed (Figure 1*A* and Supplementary Figure 1*A*–*C*, respectively) for HCs and 215 participants with complete data (moderate n = 103, severe n = 59, fatal n = 28) sampled ≤20 days after symptom onset. Clustering identified 2 groups of participants (A and B), with cluster A having more severe and fatal peak-severity participants (Figure 1*A*). Cluster A had elevated chemokines (eg, CCL2 and CCL4), inflammatory cytokines (eg, IFN-γ, IL-2, IL-6, tumor necrosis factor-α [TNF-α], IL-1β, and IL-12p70), and the anti-inflammatory cytokine IL-10 (marker cluster 1). PCA showed a concentrated group of HCs and groups of patients with COVID-19 that diverged from HCs in PC1 (explaining 50.4% of variance) and PC2 (explaining 8.1% of variance) with greater peak severity and severity at time of sampling (Supplementary Figure 1*A* and 1*B*, respectively). Loading values for PC1 and PC2 indicated IL-2, IL-12p70, TNF-α, IL-1β, and IFN-γ made strong contributions to this variance (Supplementary Figure 1*C*). These cytokines and chemokines were closely correlated, along with other markers in cluster A (Supplementary Figure 1*D*).

Despite the enrichment of patients with higher disease severity in cluster 1, this cluster included many moderate-disease patients. Similarly, cluster 2 included some fatal cases, although most had moderate disease at time of sampling (Figure 1*A*). These unsupervised analyses of mucosal cytokine and chemokine levels from patients hospitalized with COVID-19 therefore indicated proinflammatory markers were associated with higher disease severity, and that the timing of sample collection during disease may influence cytokine and chemokine levels.

### Mucosal Responses in COVID-19 Are Dominated by IFN-γ and IL-2

To define the association of individual mucosal immune markers with COVID-19 severity, levels in the first sample per participant were directly compared between peak-severity groups and HCs. This highlighted increases in a range of cytokines, including IFN-γ, IL-2, IL-12p70, IL-10, and IL-4 in moderate (n = 142), severe (n = 92), and fatal (n = 40) peak-severity groups, relative to HCs (Figure 1*B*–*D*, respectively). Analysis of individual markers demonstrated considerable overlap between peak-severity groups in many cytokines and chemokines, including IFN-γ, IL-2, and IL-10 (Figure 1*E*–*M* and Supplementary Figure 2). Levels of many markers were higher in the severe group, relative to moderate, but median levels in the fatal group were commonly equivalent to, or lower than, severe. Five markers were decreased in the moderate group, relative to HCs; angiopoietin-2, A proliferation-inducing ligand (APRIL/TNFSF13), fibroblast growth factor-basic (FGF-basic), D-dimer, and matrix metalloproteinase (MMP-1). No nasal cytokines or chemokines were significantly different between severe and fatal groups, in contrast to our previous observations in plasma [16], indicating that patients that would succumb to COVID-19 had equivalent mucosal inflammation to those that would survive severe disease.

### Equivalent Viral Loads Between Peak-Severity Groups

**Figure 2.**
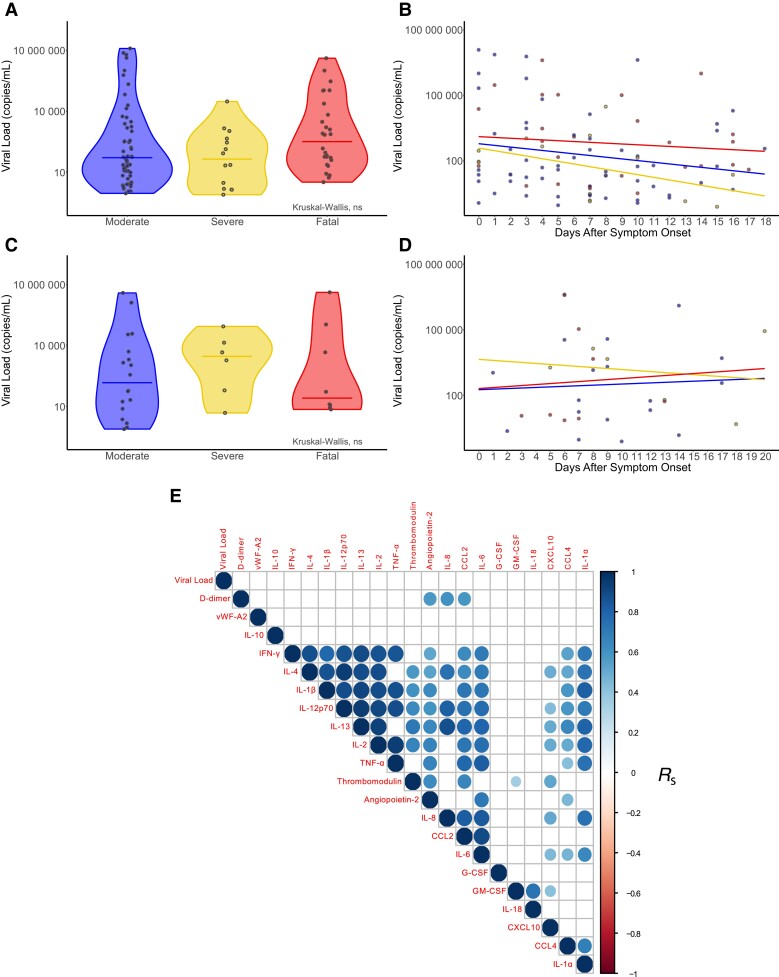
Nasal viral load was not associated with peak COVID-19 severity or nasal marker levels. *A*, Viral load quantified by RT-qPCR on nasal swabs from patients with COVID-19 (moderate n = 56, severe n = 14, fatal n = 28) taken 0–20 days after symptom onset. *B*, Viral load data aligned with the duration of symptoms at the time of sampling (days after symptom onset). A subset of these participants (moderate n = 18, severe n = 6, fatal n = 8) had viral load data and time-matched nasosorption cytokine and chemokine data: (*C*) viral load grouped by peak COVID-19 severity; and (*D*) viral load data aligned with duration of symptoms. *E*, Hierarchically clustered correlation matrix of nasal cytokines and chemokines and nasal viral loads, ranked using the Spearman rank correlation coefficient (*R*_S_). Correlations were corrected for multiple testing and nonsignificant correlations are denoted by empty boxes. *B* and *D*, Linear regression lines for moderate, severe, and fatal peak COVID-19 severity groups. Abbreviations: COVID-19, coronavirus disease 2019; G-CSF, granulocyte-colony-stimulating factor; GM-CSF, granulocyte-macrophage colony-stimulating factor; IFN-γ, interferon-γ; IL, interleukin; ns, not significant; RT-qPCR, reverse transcription quantitative polymerase chain reaction; TNF-α, tumor necrosis factor-α; vWF-A2, von Willebrand factor-A2.

We next determined whether differences in mucosal inflammation between severity groups could be accounted for by viral load. Nasal swabs collected ≤20 days after symptom onset showed equivalent viral loads between peak-severity groups (moderate n = 56, severe n = 14, fatal n = 28; Figure 2*A*). Relating these data to symptom duration at the time of sampling indicated no large-scale differences between peak-severity groups over the course of infection (Figure 2*B*). Nasal viral swabs collected at the same time as nasosorption sampling from 32 patients (n = 18 moderate, n = 6 severe, and n = 8 fatal peak severities) also showed no significant differences in viral load between severity groups (Figure 2*C*), or over time (Figure 2*D*). Viral loads also did not significantly correlate with contemporaneous nasosorption cytokine/chemokine levels (Figure 2*E*). These data indicated that viral load is unlikely to account for the differences in the scale and nature of the mucosal immune response between severity groups.

### Longitudinal Nasal Sampling Demonstrates a Limited Early Mucosal Response in Fatal COVID-19

Given the indication that severity at the time of sampling might be associated with mucosal marker levels (Figure 1*A*), we studied the kinetics of nasal cytokines and chemokines between peak-severity groups. Sequential nasal samples from 153 patients (n = 115 with 2 repeat samples; n = 34 with 3 repeat samples; n = 4 with 4 repeat samples, totaling 348 samples; Supplementary Table 2) demonstrated that median levels of IFN-γ, IL-2, IL-10, IL-12p70, IL-4, and IL-1α were low in fatal outcome participants in the first days after symptom onset, before climbing to a peak between symptom days 15 and 20 (Supplementary Figure 3). By contrast, nasal cytokine and chemokine levels in the moderate and severe peak-severity groups changed less over time.

**Figure 3.**
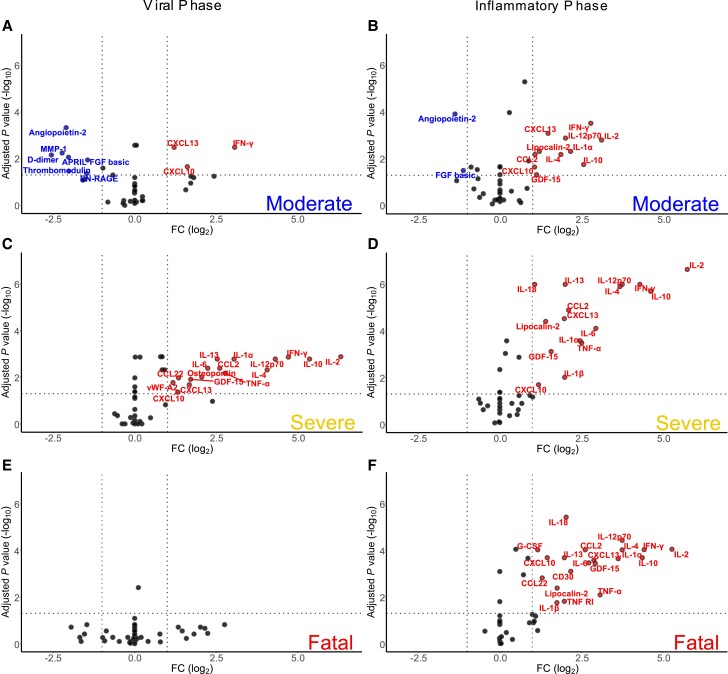
A muted early mucosal immune response was associated with fatal COVID-19. Cytokine and chemokine levels in nasal samples were compared to healthy control (n = 25) levels for each COVID-19 patient time group: (*A*) moderate, viral phase (0–5 days after symptom onset; n = 44); (*B*) moderate, inflammatory phase (6–20 days after symptom onset; n = 98); (*C*) severe, viral phase (n = 18); (*D*) severe, inflammatory phase (n = 74); (*E*) fatal, viral phase (n = 9); and (*F*) fatal, inflammatory phase (n = 31). *A*–*F*, Volcano plots with −log10 transformed false discovery rate adjusted Wilcoxon rank-sum *P* values (horizontal line at cutoff *P* ≥ .05) plotted against log_2_ fold changes relative to healthy controls (vertical lines at cutoffs ≤ -2 and ≥2 fold change). Abbreviations: APRIL, A proliferation-inducing ligand; COVID-19, coronavirus disease 2019; EN-RAGE, Extracellular newly identified receptor for advanced glycation end products/S100A12; FGF, fibroblast growth factor; GDF-15, growth differentiation factor 15; IFN-γ, interferon-γ; IL, interleukin; MMP-1, matrix metalloproteinase-1; TNF-α, tumor necrosis factor-α; vWF-A2, von Willebrand factor-A2.

**Figure 4.**
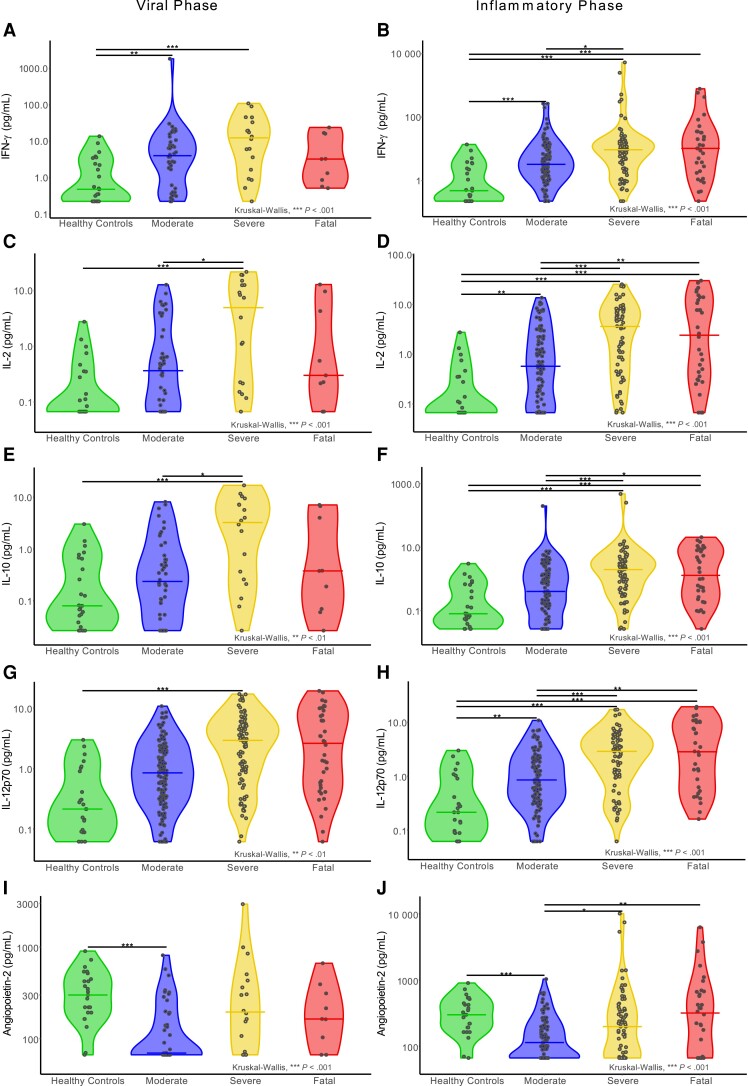
Cytokine and chemokine levels during the viral and inflammatory phases between peak-severity groups. Individual markers’ particular distinctions between each COVID-19 peak-severity group and healthy controls, displayed by viral phase (0–5 days after symptom onset) or inflammatory phase (6–20 days after symptom onset): (*A*) viral phase IFN-γ; (*B*) inflammatory phase IFN-γ; (*C*) viral phase IL-2; (*D*) inflammatory phase IL-2; (*E*) viral phase IL-10; (*F*) inflammatory phase IL-10; (*G*) viral phase IL-12p70; (*H*) inflammatory phase IL-12p70; (*I*) viral phase angiopoietin-2; and (*J*) inflammatory phase angiopoietin-2. All panels show group median levels as lines and significance levels determined using Kruskal-Wallis tests with Dunn *P* value correction. *P < .05, **P < .01, ***P < .001. Abbreviations: COVID-19, coronavirus disease 2019; IFN-γ, interferon-γ; IL, interleukin.

To determine whether cytokine and chemokine responses in fatal outcome participants were lower in the first days after symptom onset, we separated data into early (0–5 days after symptom onset, “viral”) and inflammation-dominated late (6–20 days after symptom onset “inflammatory”) phases [15]. PCA demonstrated overlap between peak-severity groups during either viral or inflammatory phases (Supplementary Figure 4). However, only the antiviral markers IFN-γ, CXCL10, and CXCL13 were elevated in the moderate group viral phase relative to HCs (Figure 3*A*), along with decreases in 7 cytokines and chemokines predominantly associated with vascular function (eg, angiopoietin-2, thrombomodulin). In the inflammatory phase, this IFN-led response expanded to include inflammatory markers (eg, IL-1α, IL-2, and IL-12p70; Figure 3*B*). A broader inflammatory state was evident during the viral phase in the severe outcome group, with IL-2, IL-10, and IFN-γ particularly increased, alongside IL-4, IL-12p70, and IL-1α (Figure 3*C*). Furthermore, IL-6 and TNF-α, previously linked to severe disease in the circulation [16], were also elevated. These changes continued in the inflammatory phase (Figure 3*D*), indicating a stronger mucosal inflammatory response in severe disease than moderate and a relatively stable nature and scale of the mucosal immune response within the severe group. In contrast to the IFN-led early mucosal responses evident in moderate and severe groups, the fatal outcome group had nasal cytokine levels equivalent to HCs in the early viral phase (Figure 3*E*). After the absence of a discernible response during the viral phase in the fatal group, the inflammatory phase was associated with induction of a wide array of cytokines and chemokines like those in the severe group (Figure 3*F*). Analyses of individual marker levels between groups in the viral and inflammatory phases supported these differences in timing and breadth of mucosal responses (Figure 4 and Supplementary Figures 5 and 6). As an exploratory analysis, we determined the ratio of viral load to mucosal CXCL10 levels (as a measure of IFN activity) where data were available from contemporaneous samples (n = 36). Stratification of these data by severity group and disease phase demonstrated lower levels (indicating low CXCL10 levels for a given viral load) in the fatal viral phase group, although this did not achieve statistical significance relative to other groups owing to the small group sizes (Supplementary Figure 7). These analyses indicated a limited mucosal response in early disease that would progress to fatal outcomes, in contrast to a largely IFN-dominated early response in those that will survive hospitalization with COVID-19, followed by a delayed but robust inflammatory response.

### Robust Peripheral Inflammation in the Early Stages of Fatal COVID-19

To determine if differences between compartments were evident, nasal data were compared to our previously reported blood data [16], selecting for plasma samples collected ≤20 days after symptom onset (n = 416), comprising moderate (n = 210), severe (n = 150), and fatal (n = 56) peak-severity groups. To align with the nasal analysis, plasma cytokine data was normalized to HCs (n = 15) and comparisons made between viral and inflammatory phases (Supplementary Figure 8 and Supplementary Table 3), demonstrating little change in the nature or scale of peripheral inflammation between early and later stages of COVID-19.

**Figure 5.**
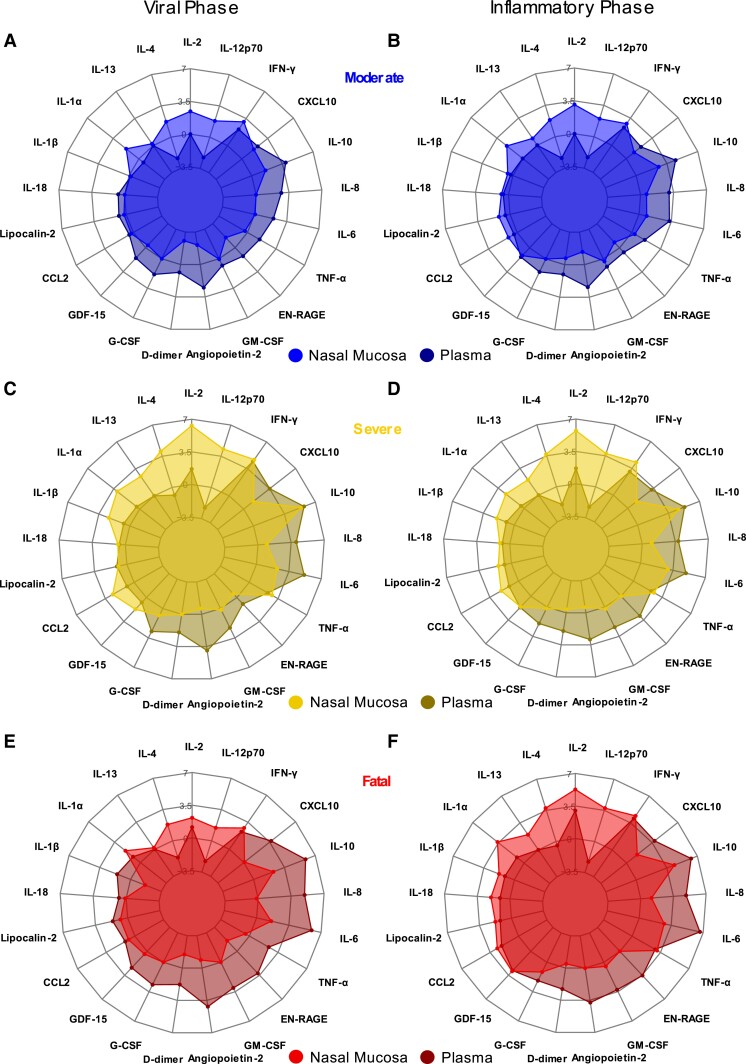
Pronounced blood plasma responses in the early stages of fatal COVID-19 despite weak mucosal responses. Median log_2_ fold-change values, relative to healthy control levels, for each cytokine or chemokine were contrasted between nasosorption samples (lighter shades) and plasma (darker shades) for each COVID-19 patient peak-severity group in viral (0–5 days after symptom onset) or inflammatory (6–20 days after symptom onset) phases: (*A*) moderate, viral phase; (*B*) moderate, inflammatory phase; (*C*) severe, viral phase; (*D*) severe, inflammatory phase; (*E*) fatal, viral phase; and (*F*) fatal, inflammatory phase. All panels show median log_2_ transformed cytokine fold-changes relative to healthy controls (healthy control levels therefore represented as the 0 line) for nasal samples and plasma samples by peak severity. Abbreviations: COVID-19, coronavirus disease 2019; EN-RAGE, Extracellular newly identified receptor for advanced glycation end products/S100A12; G-CSF, granulocyte-colony-stimulating factor; GDF-15, growth differentiation factor 15; GM-CSF, granulocyte-macrophage colony-stimulating factor; IFN-γ, interferon-γ; IL, interleukin; TNF-α, tumor necrosis factor-α.

To directly compare responses between nasal and plasma compartments, median levels of each marker in each severity group are shown as fold change relative to HC levels for each compartment (Figure 5). The viral phase of moderate participants showed prominent increases in plasma IL-10, IL-8, and angiopoietin-2, while IFN-γ and CXCL10 increases relative to HCs were similar between nasal and plasma compartments (Figure 5*A* and Supplementary Figure 8*A*). These were similar in the inflammatory phase for moderate participants (Figure 5*B* and Supplementary Figure 8*B*). In the severe group, plasma IL-6 elevations became more pronounced, during both viral and inflammatory phases (Figure 5*C* and 5*D*, respectively), while a robust elevation in nasal IL-2 was not evident in the plasma. Like the moderate group, neither nasal nor plasma cytokine and chemokine profiles in the severe group changed notably between the early viral and later inflammatory phases.

Plasma marker levels in the fatal group were similar to severe (Supplementary Figure 8); however, the nasal cytokine/chemokine response was relatively muted (Figure 5*E*). In contrast to the moderate and severe groups, the nasal marker profile in the fatal group expanded between the viral and inflammatory phases (Figure 5*F*). Interestingly, levels of IL-1β and IL-18 were elevated in nasal samples of the inflammatory phase of both severe and fatal participants but were similar to HC levels in plasma (Figures 5*D* and 5*F*).

Together, this demonstrated that IFNs and IFN-driven chemokines dominate the nasal and plasma immune response to COVID-19. In severe disease during both the viral and inflammatory phases, IL-2 dominated the response in the nose, while IL-6 increased in the plasma and IFN-γ and IFN-induced chemokines were evident in both compartments. In fatal cases during the early viral phase, the nasal immune response was diminished relative to those that would survive COVID-19; however, the plasma inflammatory response was robust.

## DISCUSSION

This is the first large-scale multicenter study of respiratory mucosal inflammation during hospitalization with COVID-19, recruiting patients early in disease. Recruitment was before SARS-CoV-2 vaccination, so reflects primary infections in naive individuals. Fatal COVID-19 was marked by a limited early mucosal immune response that contrasted with the robust IFN-led response in milder disease. This deficient mucosal response contrasted with robust inflammation in peripheral blood from the earliest stages of fatal disease. Therefore, a weak early mucosal response despite robust peripheral inflammation was associated with mortality.

Weak early mucosal IFN responses might be expected to result in elevated viral load. However, viral loads were equivalent between peak-severity groups, in agreement with larger studies [11], and were not associated with mucosal cytokine/chemokine levels. Progressive inflammatory responses in the URT may therefore represent local immune dysfunction, rather than a loss of control over viral replication. Viral clearance rates are slower in symptomatic individuals [21] and as such, longitudinal analyses of viral kinetics may more closely associate with mucosal immune responses. Self-reported symptom durations are subjective and our definition of the viral phase (0–5 days of symptoms) may be influenced by this. Pseudotime analyses using sequential viral load measurements per participant offer one opportunity to align biological data to features of viral replication that may better reflect the duration of infection. However, our viral load data were insufficient to perform such analyses, while variance in viral clearance owing to differences in immune responses can confound derivation of pseudotime durations [11, 22].

Induction of IL-2 in the mucosa across severity groups may result from local T-cell activity, potentially fitting with blood lymphopenia in severe disease due to mucosal trafficking of peripheral T cells. Unlike the blood, mucosal IFN-γ and IL-2 levels plateaued between severe and fatal COVID-19 groups in late disease. In line with these data, aberrant macrophage activation and impaired T-cell responses in diseased lungs [23], an inadequate CD8^+^ T-cell response in fatal pneumonia, poor viral regulation [24], and CD8^+^ T-cell lymphopenia are associated with severe disease [24, 25]. The early abundance of IFN-γ suggests a type I (including Th1) skewed mucosal response in milder disease, while elevations in type II cytokines like IL-13 (linked to hyaluronan synthesis and severe disease [26]) in early severe disease suggest this skewing could change in more severe disease. In contrast to differences in the early viral phase, the nasal inflammatory-phase response in the fatal group was similar in nature and scale to severe participants, suggesting that early activation of mucosal immune responses may influence disease trajectory.

Some markers were similar between compartments; however, IL-18 and IL-1β were markedly elevated in inflammatory phase nasal samples from severe and fatal groups, but not in plasma. This suggests inflammasome activation may be occurring in the airway during severe COVID-19 [27]. Nasal granulocyte-macrophage colony-stimulating factor (GM-CSF) was increased with no distinction between COVID-19 severity groups, in contrast to plasma GM-CSF (and IL-6) levels, which scale with severity [16]. Levels of mucosal IL-6 and GM-CSF therefore offer less distinction between severity groups than equivalent peripheral blood measures, highlighting common and unique features of immune responses between compartments.

A deficient IFN response has been associated with COVID-19 [28], with different mechanisms hypothesized. Firstly, the apparent lack of early IFN-centered mucosal responses in fatal disease may be attributed to age-related immunosenescence [29], which is a therapeutic target in COVID-19 [30]. This attribution is supported by the older age of our fatal outcome group, and the risk associated with older age in COVID-19 [22]. Additionally, IFN-neutralizing autoantibodies associated with severe COVID-19 [31] may stall early mucosal IFN-centered responses, encouraging progression to pathologic inflammatory responses. Expression of the IFN-induced transcript *IFI27* in peripheral blood has been linked to protection from disease in presymptomatic, asymptomatic, and abortive infections [25, 32, 33]. The weak IFN response in the mucosa may be a consequence of limited type I/III IFN expression in airway epithelial cells following SARS-CoV-2 infection [34] (despite a strong inflammatory response) and may evidence the ability of SARS-CoV-2 to repress IFN responses, but does not explain why this ability may manifest differently between severity groups when viral loads are equivalent. Levels of type I/III IFNs are low in mucosal fluid samples and were not quantifiable in our samples. Another explanation for the weak IFN response may be the abundance of polymorphisms in IFN-associated genes associated with COVID-19 [35–37], which may prevent or stall IFN responses. Together, these reports demonstrate the importance of IFN responses in limiting disease. Our data further this field by demonstrating that this deficient IFN response in the most severe cases of COVID-19 is most apparent in early mucosal responses.

The early anergic response in the nasal mucosa is reminiscent of the dysregulated immune response observed in blood during sepsis, combining elements of hyperinflammation alongside immunosuppression [38, 39]. Our data in fatal COVID-19 indicate that nasal mucosal responses are deficient even when hyperinflammation is established in blood. Such dysregulation was also apparent as inflammasome activation in our data, suggesting that severe COVID-19 is associated with failure to mount early IFN responses in the mucosa, alongside epithelial cell damage and accumulation of activated macrophages that result in delayed but excessive cytokine release [34, 40].

Our study is the largest to profile mucosal immune responses during severe COVID-19. Despite this scale, our sample size is limited in places, such as the number of longitudinal samples during early disease that progressed to fatal outcome. Respiratory sampling is challenging in patients with severe disease, resulting in the paucity of large-scale studies of mucosal responses in COVID-19, relative to the number of studies on blood responses. These difficulties were mitigated through our use of minimally invasive nasosorption sampling [41]. However, the URT cannot be assumed to fully represent the lower airway. As such, our observation of delayed IFN responses in fatal COVID-19 may not be recapitulated in the lower airway. Lower airway viral replication may similarly differ between severity groups in a way not evident in the URT; however, obtaining lower airway samples from milder cases of COVID-19 is challenging and sputum and nasal viral loads correlate closely [42].

Our viral and inflammatory phases could be considered arbitrary; however, we selected these time period classifications as they have been suggested to define the point at which initial antiviral responses transition to inflammatory responses [15]. Our cohort were recruited prior to vaccine rollout and were unlikely to have been previously infected, given our recruitment in early 2020. Vaccination, SARS-CoV-2 infection history, and viral evolution are all likely to change cytokine, chemokine, and immune cell responses [43], and our findings should be validated in a modern cohort.

Immune suppression with corticosteroids and monoclonal antibodies benefit patients with COVID-19 [6, 7]. Peripheral IFN-λ administration has not achieved widespread therapeutic use [13]. Similarly, nebulization of IFN-β to the airway showed promise in phase 2 trials [44] but phase 3 trials recently failed to meet primary end points, despite showing promise in preventing disease progression [45]. Our data indicate that the window of opportunity for correcting mucosal IFN deficiencies may be in the first days of symptoms, so targeting such therapies to this window may enhance apparent efficacy. The RECOVERY trials of dexamethasone demonstrated greatest benefit >7 days after symptom onset [6], roughly aligning with our transition between viral and inflammatory phases. Similarly, anti–IL-6 is most effective in patients with elevated C-reactive protein [7], supporting the need to target therapies to distinct stages and phenotypes of disease.

We studied mucosal samples from SARS-CoV-2–naive participants hospitalized early in the pandemic. These unique features enabled us to demonstrate early IFN-related mucosal immune responses in hospitalized COVID-19 patients who survived, that were absent in those that progressed to fatal disease. These data highlight the importance of early antiviral responses in the mucosa in protecting against severe COVID-19 and may guide the development and testing of therapeutics for the respiratory mucosa.

## Supplementary Data

Supplementary materials are available at *The Journal of Infectious Diseases* online (http://jid.oxfordjournals.org/). Supplementary materials consist of data provided by the author that are published to benefit the reader. The posted materials are not copyedited. The contents of all supplementary data are the sole responsibility of the authors. Questions or messages regarding errors should be addressed to the author.

## Supplementary Material

jiad590_Supplementary_Data

## Notes

***Acknowledgments.*** The ISARIC4C Investigators: Co-Lead Investigator (Consortium Lead and joint CI): J Kenneth Baillie. Co-Lead Investigator (Imperial College): Peter J. M. Openshaw. Colead Investigator (Protocol CI): Malcolm G. Semple. Coinvestigator: Beatrice Alex, Petros Andrikopoulos, Benjamin Bach, Wendy S. Barclay, Debby Bogaert, Meera Chand, Kanta Chechi, Graham S. Cooke, Ana da Silva Filipe, Thushan de Silva, Annemarie B. Docherty, Gonçalo dos Santos Correia, Marc-Emmanuel Dumas, Jake Dunning, Tom Fletcher, Christoper A. Green, William Greenhalf, Julian L. Griffin, Rishi K. Gupta, Ewen M. Harrison, Antonia Ying Wai Ho, Karl Holden, Peter W. Horby, Samreen Ijaz, Saye Khoo, Paul Klenerman, Andrew Law, Matthew R. Lewis, Sonia Liggi, Wei Shen Lim, Lynn Maslen, Alexander J. Mentzer, Laura Merson, Alison M. Meynert, Shona C. Moore, Mahdad Noursadeghi, Michael Olanipekun, Anthonia Osagie, Massimo Palmarini, Carlo Palmieri, William A. Paxton, Georgios Pollakis, Nicholas Price, Andrew Rambaut, David L. Robertson, Clark D. Russell, Vanessa Sancho-Shimizu, Caroline J. Sands, Janet T. Scott, Louise Sigfrid, Tom Solomon, Shiranee Sriskandan, David Stuart, Charlotte Summers, Olivia V. Swann, Zoltan Takats, Panteleimon Takis, Richard S. Tedder, A. A. Roger Thompson, Emma C. Thomson, Ryan S. Thwaites, Lance C. W. Turtle, Maria Zambon. Data Analyst: Thomas M. Drake, Cameron J. Fairfield, Stephen R. Knight, Kenneth A. Mclean, Derek Murphy, Lisa Norman, Riinu Pius, Catherine A. Shaw. Data and Information System Manager: Marie Connor, Jo Dalton, Carrol Gamble, Michelle Girvan, Sophie Halpin, Janet Harrison, Clare Jackson, James Lee, Laura Marsh, Daniel Plotkin, Stephanie Roberts, Egle Saviciute. Data Integration and Presentation: Sara Clohisey, Ross Hendry, Susan Knight, Eva Lahnsteiner, Andrew Law, Gary Leeming, Lucy Norris, James Scott-Brown, Sarah Tait, Murray Wham. ISARIC Clinical Coordinator: Gail Carson. Edinburgh Laboratory Staff and Volunteers: Richard Clark, Audrey Coutts, Lorna Donnelly, Angie Fawkes, Tammy Gilchrist, Katarzyna Hafezi, Louise MacGillivray, Alan Maclean, Sarah McCafferty, Kirstie Morrice, Lee Murphy, Nicola Wrobel. Local Principal Investigators: Kayode Adeniji, Daniel Agranoff, Ken Agwuh, Dhiraj Ail, Erin L. Aldera, Ana Alegria, Sam Allen, Brian Angus, Abdul Ashish, Dougal Atkinson, Shahedal Bari, Gavin Barlow, Stella Barnass, Nicholas Barrett, Christopher Bassford, Sneha Basude, David Baxter, Michael Beadsworth, Jolanta Bernatoniene, John Berridge, Colin Berry, Nicola Best, Pieter Bothma, Robin Brittain-Long, Naomi Bulteel, Tom Burden, Andrew Burtenshaw, Vikki Caruth, David Chadwick, David Chadwick, Duncan Chambler, Nigel Chee, Jenny Child, Srikanth Chukkambotla, Tom Clark, Paul Collini, Catherine Cosgrove, Jason Cupitt, Maria-Teresa Cutino-Moguel, Paul Dark, Chris Dawson, Samir Dervisevic, Phil Donnison, Sam Douthwaite, Andrew Drummond, Ingrid DuRand, Ahilanadan Dushianthan, Tristan Dyer, Cariad Evans, Chi Eziefula, Chrisopher Fegan, Adam Finn, Duncan Fullerton, Sanjeev Garg, Atul Garg, Effrossyni Gkrania-Klotsas, Jo Godden, Arthur Goldsmith, Clive Graham, Tassos Grammatikopoulos, Elaine Hardy, Stuart Hartshorn, Daniel Harvey, Peter Havalda, Daniel B. Hawcutt, Maria Hobrok, Luke Hodgson, Anil Hormis, Joanne Howard, Michael Jacobs, Susan Jain, Paul Jennings, Agilan Kaliappan, Vidya Kasipandian, Stephen Kegg, Michael Kelsey, Jason Kendall, Caroline Kerrison, Ian Kerslake, Oliver Koch, Gouri Koduri, George Koshy, Shondipon Laha, Steven Laird, Susan Larkin, Tamas Leiner, Patrick Lillie, James Limb, Vanessa Linnett, Jeff Little, Mark Lyttle, Michael MacMahon, Emily MacNaughton, Ravish Mankregod, Huw Masson, Elijah Matovu, Katherine McCullough, Ruth McEwen, Manjula Meda, Gary Mills, Jane Minton, Kavya Mohandas, Quen Mok, James Moon, Elinoor Moore, Patrick Morgan, Craig Morris, Katherine Mortimore, Samuel Moses, Mbiye Mpenge, Rohinton Mulla, Michael Murphy, Thapas Nagarajan, Megan Nagel, Mark Nelson, Lillian Norris, Matthew K. O'Shea, Marlies Ostermann, Igor Otahal, Mark Pais, Carlo Palmieri, Selva Panchatsharam, Danai Papakonstantinou, Padmasayee Papineni, Hassan Paraiso, Brij Patel, Natalie Pattison, Justin Pepperell, Mark Peters, Mandeep Phull, Stefania Pintus, Tim Planche, Frank Post, David Price, Rachel Prout, Nikolas Rae, Henrik Reschreiter, Tim Reynolds, Neil Richardson, Mark Roberts, Devender Roberts, Alistair Rose, Guy Rousseau, Bobby Ruge, Brendan Ryan, Taranprit Saluja, Sarah Cole, Matthias L. Schmid, Aarti Shah, Manu Shankar-Hari, Prad Shanmuga, Anil Sharma, Anna Shawcross, Jagtur Singh Pooni, Jeremy Sizer, Richard Smith, Catherine Snelson, Nick Spittle, Nikki Staines, Tom Stambach, Richard Stewart, Pradeep Subudhi, Tamas Szakmany, Kate Tatham, Jo Thomas, Chris Thompson, Robert Thompson, Ascanio Tridente, Darell Tupper-Carey, Mary Twagira, Nick Vallotton, Rama Vancheeswaran, Rachel Vincent, Lisa Vincent-Smith, Shico Visuvanathan, Alan Vuylsteke, Sam Waddy, Rachel Wake, Andrew Walden, Ingeborg Welters, Tony Whitehouse, Paul Whittaker, Ashley Whittington, Meme Wijesinghe, Martin Williams, Lawrence Wilson, Stephen Winchester, Martin Wiselka, Adam Wolverson, Daniel G. Wootton, Andrew Workman, Bryan Yates, Peter Young. Material Management: Sarah E. McDonald, Victoria Shaw. Outbreak Laboratory Staff and Volunteers: Katie A. Ahmed, Jane A. Armstrong, Milton Ashworth, Innocent G. Asiimwe, Siddharth Bakshi, Samantha L. Barlow, Laura Booth, Benjamin Brennan, Katie Bullock, Nicola Carlucci, Emily Cass, Benjamin WA Catterall, Jordan J. Clark, Emily A. Clarke, Sarah Cole, Louise Cooper, Helen Cox, Christopher Davis, Oslem Dincarslan, Alejandra Doce Carracedo, Chris Dunn, Philip Dyer, Angela Elliott, Anthony Evans, Lorna Finch, Lewis W. S. Fisher, Lisa Flaherty, Terry Foster, Isabel Garcia-Dorival, Philip Gunning, Catherine Hartley, Anthony Holmes, Rebecca L. Jensen, Christopher B. Jones, Trevor R. Jones, Shadia Khandaker, Katharine King, Robyn T. Kiy, Chrysa Koukorava, Annette Lake, Suzannah Lant, Diane Latawiec, Lara Lavelle-Langham, Daniella Lefteri, Lauren Lett, Lucia A. Livoti, Maria Mancini, Hannah Massey, Nicole Maziere, Sarah McDonald, Laurence McEvoy, John McLauchlan, Soeren Metelmann, Nahida S. Miah, Joanna Middleton, Joyce Mitchell, Shona C. Moore, Ellen G. Murphy, Rebekah Penrice-Randal, Jack Pilgrim, Tessa Prince, Will Reynolds, P. Matthew Ridley, Debby Sales, Victoria E. Shaw, Rebecca K. Shears, Benjamin Small, Krishanthi S. Subramaniam, Agnieska Szemiel, Aislynn Taggart, Jolanta Tanianis-Hughes, Jordan Thomas, Erwan Trochu, Libby van Tonder, Eve Wilcock, J. Eunice Zhang. Patient Engagement: Seán Keating. Project Administrator: Cara Donegan, Rebecca G. Spencer, Primrose Chikowore. Project Manager: Chloe Donohue, Fiona Griffiths, Hayley Hardwick, Wilna Oosthuyzen.

***Author contributions*.** J. K. S., A. S. S. U., and R. S. T. performed experiments, collected experimental data, analyzed data, and performed statistical analysis. M. K. S., F. L., and A. S. S. U. contributed to sample and data collection and coordination. C. D. R., S. C. M., H. E. H., W. O., L. T., and J. K. B. provided clinical data and oversaw study participant recruitment. C. D. and E. C. T. performed viral load quantification and generated viral load data. M. G. S., J. K. B., and P. J. M. O. designed and delivered the ISARIC4C consortium project. The study was designed by R. S. T. and P. J. M. O. The manuscript was written by J. K. S., R. S. T., and P. J. M. O. All authors contributed with critical revisions and approved the final manuscript.

***Disclaimer*.** The views expressed are those of the authors and not necessarily those of the National Institute for Health Research, Bill and Melinda Gates Foundation, US Food and Drug Administration, Medical Research Council, Wellcome Trust, or Public Health England.

***Financial support*.** This work was supported by the National Institute for Health Research (NIHR) (grant number CO-CIN-01); the UK Research and Innovation (UKRI) Medical Research Council (MRC) (grant numbers MC_PC_19059 and MC_UU_00034/6); NIHR Health Protection Research Senior Investigator Award (to P. J. M. O.); the NIHR Imperial Biomedical Research Centre (grant number P45058); the Health Protection Research Unit (HPRU) in Respiratory Infections at Imperial College London and NIHR HPRU in Emerging and Zoonotic Infections at University of Liverpool, both in partnership with Public Health England, NIHR (grant number 200907); Wellcome Trust and Department for International Development (grant number 215091/Z/18/Z); the Bill and Melinda Gates Foundation (grant number OPP1209135); and the UK Coronavirus Immunology Consortium. Liverpool Experimental Cancer Medicine Centre provided infrastructure support (grant number C18616/A25153). L. T. is supported by the Wellcome Trust (grant number 205228/Z/16/Z) and by US Food and Drug Administration Medical Countermeasures Initiative (grant number 75F40120C00085). F. L. is supported by an MRC Clinical Training Fellowship (grant number MR/W000970/1). Funding to pay the Open Access publication charges for this article was provided by a Jisc Read and Publish deal for UK institutions, including Imperial College London.

## References

[1] Chiu C , Openshaw PJ. Antiviral B cell and T cell immunity in the lungs. Nat Immunol 2015; 16:18–26. 25521681 10.1038/ni.3056 PMC7097128

[2] Akamatsu MA , de Castro JT, Takano CY, Ho PL. Off balance: interferons in COVID-19 lung infections. EBioMedicine 2021; 73:103642. 34678609 10.1016/j.ebiom.2021.103642 PMC8524139

[3] Diamond MS , Kanneganti TD. Innate immunity: the first line of defense against SARS-CoV-2. Nat Immunol 2022; 23:165–76. 35105981 10.1038/s41590-021-01091-0 PMC8935980

[4] Sette A , Crotty S. Adaptive immunity to SARS-CoV-2 and COVID-19. Cell 2021; 184:861–80. 33497610 10.1016/j.cell.2021.01.007 PMC7803150

[5] Nielsen SS , Vibholm LK, Monrad I, et al SARS-CoV-2 elicits robust adaptive immune responses regardless of disease severity. EBioMedicine 2021; 68:103410. 34098342 10.1016/j.ebiom.2021.103410 PMC8176920

[6] Horby P , Lim WS, Emberson JR, et al Dexamethasone in hospitalized patients with COVID-19. New Engl J Med 2021; 384:693–704. 32678530 10.1056/NEJMoa2021436 PMC7383595

[7] RECOVERY Collaborative Group . Tocilizumab in patients admitted to hospital with COVID-19 (RECOVERY): a randomised, controlled, open-label, platform trial. Lancet 2021; 397:1637–45. 33933206 10.1016/S0140-6736(21)00676-0 PMC8084355

[8] Butler CC , Hobbs FDR, Gbinigie OA, et al Molnupiravir plus usual care versus usual care alone as early treatment for adults with COVID-19 at increased risk of adverse outcomes (PANORAMIC): an open-label, platform-adaptive randomised controlled trial. Lancet 2023; 401:281–93. 36566761 10.1016/S0140-6736(22)02597-1 PMC9779781

[9] Gupta A , Gonzalez-Rojas Y, Juarez E, et al Early treatment for COVID-19 with SARS-CoV-2 neutralizing antibody sotrovimab. N Engl J Med 2021; 385:1941–50. 34706189 10.1056/NEJMoa2107934

[10] Smith N , Goncalves P, Charbit B, et al Distinct systemic and mucosal immune responses during acute SARS-CoV-2 infection. Nat Immunol 2021; 22:1428–39. 34471264 10.1038/s41590-021-01028-7 PMC8553615

[11] Challenger JD , Foo CY, Wu Y, et al Modelling upper respiratory viral load dynamics of SARS-CoV-2. BMC Med 2022; 20:25. 35022051 10.1186/s12916-021-02220-0 PMC8755404

[12] Fajnzylber J , Regan J, Coxen K, et al SARS-CoV-2 viral load is associated with increased disease severity and mortality. Nat Commun 2020; 11:5493. 33127906 10.1038/s41467-020-19057-5 PMC7603483

[13] Reis G , Moreira Silva EAS, Medeiros Silva DC, et al Early treatment with pegylated interferon lambda for COVID-19. N Engl J Med 2023; 388:518–28. 36780676 10.1056/NEJMoa2209760 PMC9933926

[14] Wu Y , Kang L, Guo Z, Liu J, Liu M, Liang W. Incubation period of COVID-19 caused by unique SARS-CoV-2 strains: a systematic review and meta-analysis. JAMA Netw Open 2022; 5:e2228008. 35994285 10.1001/jamanetworkopen.2022.28008 PMC9396366

[15] Openshaw PJM . Using correlates to accelerate vaccinology. Science 2022; 375:22–3. 34990231 10.1126/science.abn0007

[16] Thwaites RS , Sanchez Sevilla Uruchurtu A, Siggins MK, et al Inflammatory profiles across the spectrum of disease reveal a distinct role for GM-CSF in severe COVID-19. Sci Immunol 2021; 6:eabg9873. 33692097 10.1126/sciimmunol.abg9873 PMC8128298

[17] Dunning JW , Merson L, Rohde GGU, et al Open source clinical science for emerging infections. Lancet Infect Dis 2014; 14:8–9. 24355025 10.1016/S1473-3099(13)70327-X PMC7158987

[18] ISARIC4C. 2020. isaric4c.net/samples_access/. Accessed 11 January 2024.

[19] WHO Working Group on the Clinical Characterisation and Management of COVID-19 Infection . A minimal common outcome measure set for COVID-19 clinical research. Lancet Infect Dis 2020; 20:e192–e7. 32539990 10.1016/S1473-3099(20)30483-7 PMC7292605

[20] Docherty AB , Harrison EM, Green CA, et al Features of 20 133 UK patients in hospital with COVID-19 using the ISARIC WHO clinical characterisation protocol: prospective observational cohort study. BMJ 2020; 369:m1985. 32444460 10.1136/bmj.m1985 PMC7243036

[21] Kissler SM , Fauver JR, Mack C, et al Viral dynamics of acute SARS-CoV-2 infection and applications to diagnostic and public health strategies. PLoS Biol 2021; 19:e3001333. 34252080 10.1371/journal.pbio.3001333 PMC8297933

[22] Moradi Marjaneh M , Challenger JD, Salas A, et al Analysis of blood and nasal epithelial transcriptomes to identify mechanisms associated with control of SARS-CoV-2 viral load in the upper respiratory tract. J Infect 2023; 87:538–50. 37863321 10.1016/j.jinf.2023.10.009

[23] Melms JC , Biermann J, Huang H, et al A molecular single-cell lung atlas of lethal COVID-19. Nature 2021; 595:114–9. 33915568 10.1038/s41586-021-03569-1 PMC8814825

[24] Shi H , Wang W, Yin J, et al The inhibition of IL-2/IL-2R gives rise to CD8^+^ T cell and lymphocyte decrease through JAK1-STAT5 in critical patients with COVID-19 pneumonia. Cell Death Dis 2020; 11:429. 32513989 10.1038/s41419-020-2636-4 PMC7276960

[25] Chandran A , Rosenheim J, Nageswaran G, et al Rapid synchronous type 1 IFN and virus-specific T cell responses characterize first wave non-severe SARS-CoV-2 infections. Cell Rep Med 2022; 3:100557. 35474751 10.1016/j.xcrm.2022.100557 PMC8895494

[26] Donlan AN , Sutherland TE, Marie C, et al IL-13 is a driver of COVID-19 severity. JCI Insight 2021; 6:e150107. 34185704 10.1172/jci.insight.150107 PMC8410056

[27] Winsor N , Krustev C, Bruce J, Philpott DJ, Girardin SE. Canonical and noncanonical inflammasomes in intestinal epithelial cells. Cell Microbiol 2019; 21:e13079. 31265745 10.1111/cmi.13079

[28] Hadjadj J , Yatim N, Barnabei L, et al Impaired type I interferon activity and inflammatory responses in severe COVID-19 patients. Science 2020; 369:718–24. 32661059 10.1126/science.abc6027 PMC7402632

[29] Santoro A , Bientinesi E, Monti D. Immunosenescence and inflammaging in the aging process: age-related diseases or longevity? Ageing Res Rev 2021; 71:101422. 34391943 10.1016/j.arr.2021.101422

[30] Cox LS , Bellantuono I, Lord JM, et al Tackling immunosenescence to improve COVID-19 outcomes and vaccine response in older adults. Lancet Healthy Longev 2020; 1:e55–e7. 33521768 10.1016/S2666-7568(20)30011-8 PMC7834195

[31] Bastard P , Rosen LB, Zhang Q, et al Autoantibodies against type I IFNs in patients with life-threatening COVID-19. Science 2020; 370:eabd4585. 32972996 10.1126/science.abd4585 PMC7857397

[32] Gupta RK , Rosenheim J, Bell LC, et al Blood transcriptional biomarkers of acute viral infection for detection of pre-symptomatic SARS-CoV-2 infection: a nested, case-control diagnostic accuracy study. Lancet Microbe 2021; 2:e508–e17. 34250515 10.1016/S2666-5247(21)00146-4 PMC8260104

[33] Swadling L , Diniz MO, Schmidt NM, et al Pre-existing polymerase-specific T cells expand in abortive seronegative SARS-CoV-2. Nature 2022; 601:110–7. 34758478 10.1038/s41586-021-04186-8 PMC8732273

[34] Blanco-Melo D , Nilsson-Payant BE, Liu WC, et al Imbalanced host response to SARS-CoV-2 drives development of COVID-19. Cell 2020; 181:1036–45.e9. 32416070 10.1016/j.cell.2020.04.026 PMC7227586

[35] COVID-19 Host Genetics Initiative . Mapping the human genetic architecture of COVID-19. Nature 2021; 600:472–7. 34237774 10.1038/s41586-021-03767-x PMC8674144

[36] COVID-19 Host Genetics Initiative . A first update on mapping the human genetic architecture of COVID-19. Nature 2022; 608:E1–E10. 35922517 10.1038/s41586-022-04826-7 PMC9352569

[37] Pairo-Castineira E , Rawlik K, Bretherick AD, et al GWAS and meta-analysis identifies 49 genetic variants underlying critical COVID-19. Nature 2023; 617:764–8. 37198478 10.1038/s41586-023-06034-3 PMC10208981

[38] Torres LK , Pickkers P, van der Poll T. Sepsis-induced immunosuppression. Annu Rev Physiol 2022; 84:157–81. 34705481 10.1146/annurev-physiol-061121-040214

[39] van der Poll T , Shankar-Hari M, Wiersinga WJ. The immunology of sepsis. Immunity 2021; 54:2450–64. 34758337 10.1016/j.immuni.2021.10.012

[40] Galani IE , Rovina N, Lampropoulou V, et al Untuned antiviral immunity in COVID-19 revealed by temporal type I/III interferon patterns and flu comparison. Nat Immunol 2021; 22:32–40. 33277638 10.1038/s41590-020-00840-x

[41] Thwaites R , Jarvis H, Singh N, et al Absorption of nasal and bronchial fluids: precision sampling of the human respiratory mucosa and laboratory processing of samples. J Vis Exp 2018; 131:e56413. 10.3791/56413 PMC5908664 29443104

[42] Pan Y , Zhang D, Yang P, Poon LLM, Wang Q. Viral load of SARS-CoV-2 in clinical samples. Lancet Infect Dis 2020; 20:411–2. 32105638 10.1016/S1473-3099(20)30113-4 PMC7128099

[43] Bertoletti A , Le Bert N, Tan AT. SARS-CoV-2-specific T cells in the changing landscape of the COVID-19 pandemic. Immunity 2022; 55:1764–78. 36049482 10.1016/j.immuni.2022.08.008 PMC9385766

[44] Monk PD , Marsden RJ, Tear VJ, et al Safety and efficacy of inhaled nebulised interferon beta-1a (SNG001) for treatment of SARS-CoV-2 infection: a randomised, double-blind, placebo-controlled, phase 2 trial. Lancet Respir Med 2021; 9:196–206. 33189161 10.1016/S2213-2600(20)30511-7 PMC7836724

[45] Monk PD , Brookes JL, Tear VJ, et al Nebulised interferon-beta1a (SNG001) in hospitalised COVID-19: sPRINTER phase III study. ERJ Open Res 2023; 9:00605-2022. 36994453 10.1183/23120541.00605-2022 PMC9790107

