# Supplementary material for: Delayed Mucosal Antiviral Responses Despite Robust Peripheral Inflammation in Fatal COVID-19
Source: J Infect Dis. 2023 Dec 22;230(1):e17–29. doi: 10.1093/infdis/jiad590 (PMC11272059; doi:10.1093/infdis/jiad590)
Supplement: jiad590_Supplementary_Data [file jiad590_supplementary_data.zip › JID ISARIC Mucosal Mediators_SuppMaterials_short_R1.docx]

**Supplementary Methods**

Study registration and approvals

The ISARIC WHO CCP-UK study was registered at https://www.isrctn.com/ISRCTN66726260 and designated an Urgent Public Health Research Study by the National Institute for Health Research UK. Ethical approval for the ISARIC WHO CCP-UK and this work was given by the South Central - Oxford C Research Ethics Committee in England (Ref 13/SC/0149), the Scotland A Research Ethics Committee (Ref 20/SS/0028), and the WHO Ethics Review Committee (RPC571 and RPC572, 25 April 2013). Healthy controls were recruited prior to December 2019 under approval from the London – Harrow Research Ethics Committee (13/LO/1899) or from healthy donors following informed consent from a sub-collection of the Imperial College Healthcare NHS Trust National Institute for Health Research Imperial Biomedical Research Centre Tissue Bank. Use of this sub-collection was approved by the Tissue Bank Ethics Committee (Approval R12023).

Clinical data collection

Participants were recruited to the study between March and November 2020 (prior to the roll-out of SARS-CoV-2 vaccines in the UK). Data on patient characteristics, treatments received in hospital and outcomes were collected utilising a prespecified case report form. A modified Charlson comorbidity index was used to define comorbidities and obesity was clinician-defined. COVID-19 severity was assessed according to the World Health Organization COVID-19 ordinal scale for clinical improvement [19]. Data were available to report a patient’s peak illness severity using this scale.

Nasosorption Sample Collection and Processing

Hospitalised patients with confirmed COVID-19 were recruited to the ISARIC4C study enabling the collection of nasosorption samples as previously described [20]. Nasosorption samples were returned to their container tubes after collection and stored at -80°C for shipment to a central processing laboratory. SAM strips were thawed and elutions performed as previously described, with the modification of the assay buffer to contain 1% Triton-X100 to ensure SARS-CoV-2 inactivation [20]. Eluted aliquots were stored at -80°C until immunoassay.

Cytokine Measurements

Quantification of Angiopoietin-2, APRIL, CCL2, CCL4, CCL11, CCL22, CXCL10, CXCL13, D-dimer, EN-RAGE (S100A12), FGF basic, G-CSF, GDF-15, GM-CSF, IL-1α, IL-7, IL-18, Lipocalin-2, MMP-1, Osteopontin, Pentraxin 3, Thrombomodulin, TNF RI, VEGF and vWF-A2 were achieved with a Bio Plex 200 instrument (Bio-Rad, Hercules, California, USA) and custom Luminex panel kits (Biotechne, Minneapolis, Minnesota, USA). IFNγ, IL-10, IL-12p70, IL-13, IL-1β, IL-2, IL-4, IL-6, IL-8 and TNF-α were quantified utilising the MSD (Mesoscale Diagnostics, Rockville, Maryland, USA) V-plex proinflammatory panel on a SQ120 Quickplex instrument. Values below the lower limit of detection (LLOD) or above the upper limit of detection (ULOD) were replaced with the geometric mean of either the lower or upper detection limits, respectively, across plates for each assay.

Viral Load measurements

Viral load measurements were carried out as previously described [21]. Viral RNA was extracted from nasopharyngeal (NP) or combined NP/throat swabs supernatants using the RNAdvance blood kit (Beckman Coulter Life Sciences) following manufacturer’s recommendations. RT-qPCR was carried out using a Luna Universal Probe one-step RT-qPCR kit (New England Biolabs, E3006E). SARS-CoV-2-specific RNAs were detected by targeting the N gene from the Centres for Disease Control and Prevention panel as part of the SARS-CoV-2 Research Use Only qPCR Probe kit (Integrated DNA Technologies) using the following set of primers and probes: SARS-CoV-2_N1_Forward 5’ GACCCCAAAATCAGCGAAAT 3’, SARS-CoV-2 N1_Reverse 5’ TCTGGTTACTGCCAGTTGAATCTG 3’, SARS-CoV-2_N1_Probe ACCCCGCATTACGTTTGGTGGACC with FAM/BHQ1 modifications. SARS-CoV-2 RNA was used to generate a standard curve, and viral genomes were quantified and expressed as number of N RNA molecules per ml of supernatant. All runs were performed on the ABI7500 Fast instrument and results analysed with the 7500 Software v2.3 (Applied Biosystems, Life Technologies).

Statistical Analysis

Data were analysed and plots created utilising R Studio version 4.2.2 on the RStudio platform. Data were log_10_ transformed, scaled and centred prior to heatmap generation with the *ComplexHeatmap* package and before PCA analysis with the *prcomp* function. Plots were generated using the *fviz* function within the *factoextra* package. Optimal cluster determination limited K-means clustering to 2 clusters (total within sum of squares versus number of clusters) and PCA plots were limited to the first two PCs (explained variance versus PCs) which explained the most variance within our data. Correlograms were created using the *corrplot* function with violin, volcano, scatter and spaghetti plots generated utilising the *ggplot2* package. Radar plots were created with the *FMSB* package. Individual mediator levels relating to peak severity were tested with Kruskal-Wallis one-way analysis of variance and Dunn’s test for post hoc pairwise analysis. Volcano plot testing used the Wilcoxon Rank Sum test.

**Supplementary Figures**


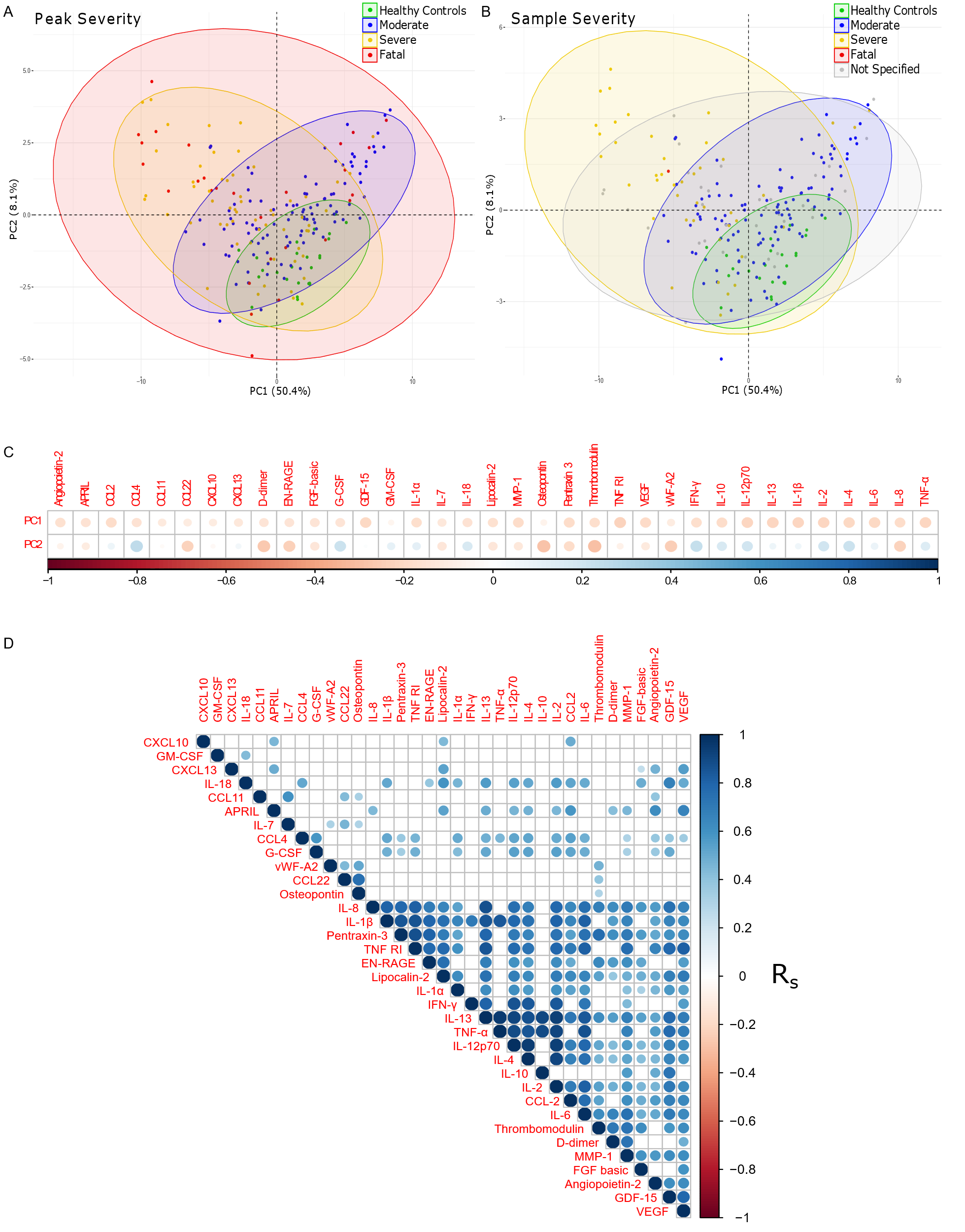


**Supplementary Figure 1 – Severe and Fatal COVID-19 deviates from Moderate and healthy control groups due to anti-viral and inflammatory cytokines**

Principal component analysis (PCA) visualisation of nasal mediator levels in patients with COVID-19 (n=274) annotated by A) patient peak severity or B) disease severity at the time of sample collection. C) PCA loading values, showing those mediators that have the most influence on variation across PC1 and PC2. D) Hierarchically clustered correlation matrix of nasal mediator levels in all COVID-19 patient samples, ranked using Spearman’s rank correlation coefficient. Blank spaces in panel D denote correlations that are not significant, after p-value correction for multiple testing.


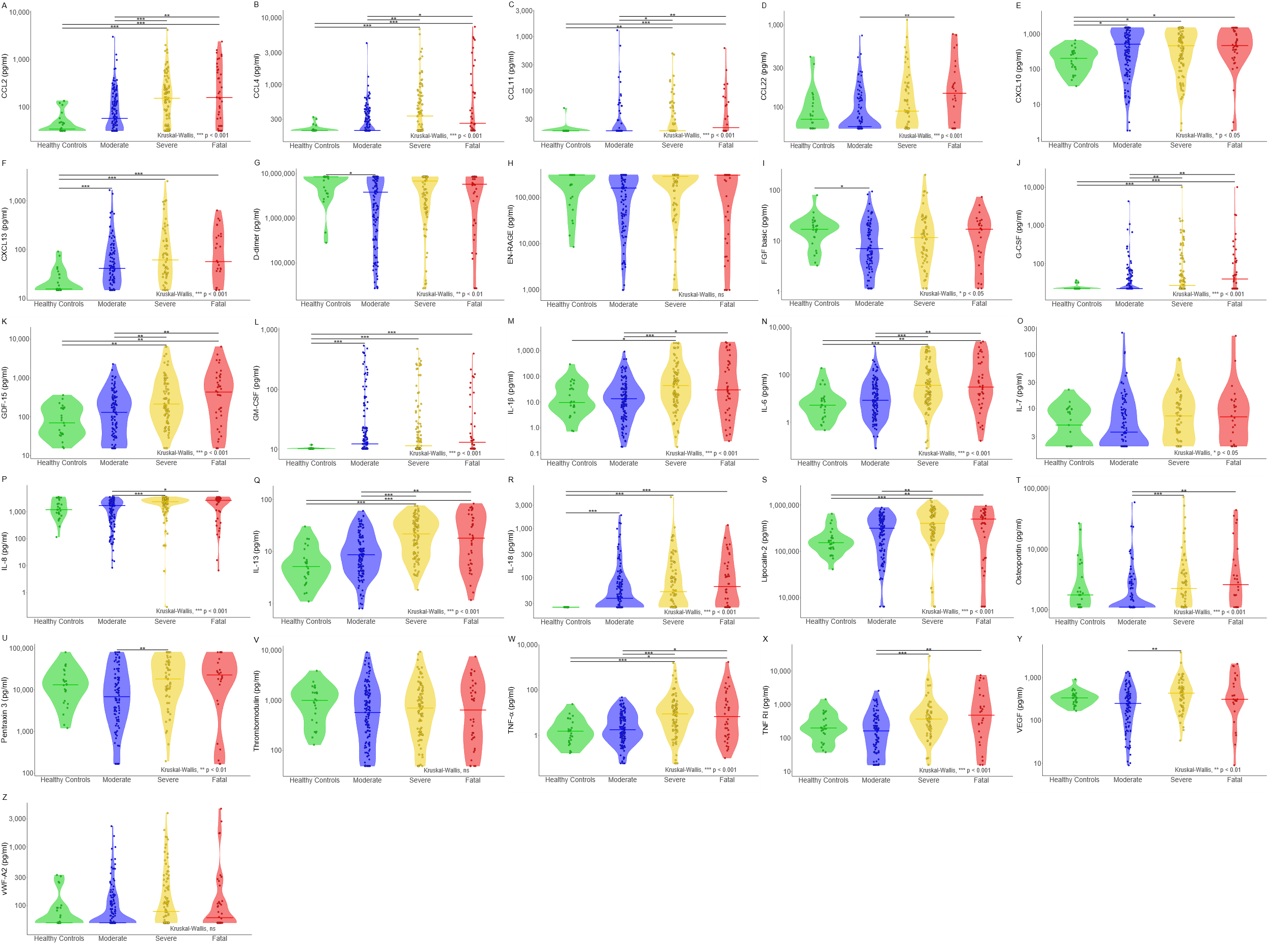


**Supplementary Figure 2 – Mediator levels in nasosorption samples from patients with COVID-19 and healthy controls**

Nasosorption samples were collected from patients during hospitalisation with COVID-19. Samples were grouped according to peak disease severity; Moderate (n=142), Severe (n=92) and Fatal (n=40). Immune mediators are ordered alphabetically. Statistical significance was tested using Kruskal-Wallis tests with Dunn’s p-value correction. *P<0.05, **P<0.01, ***P<0.001.


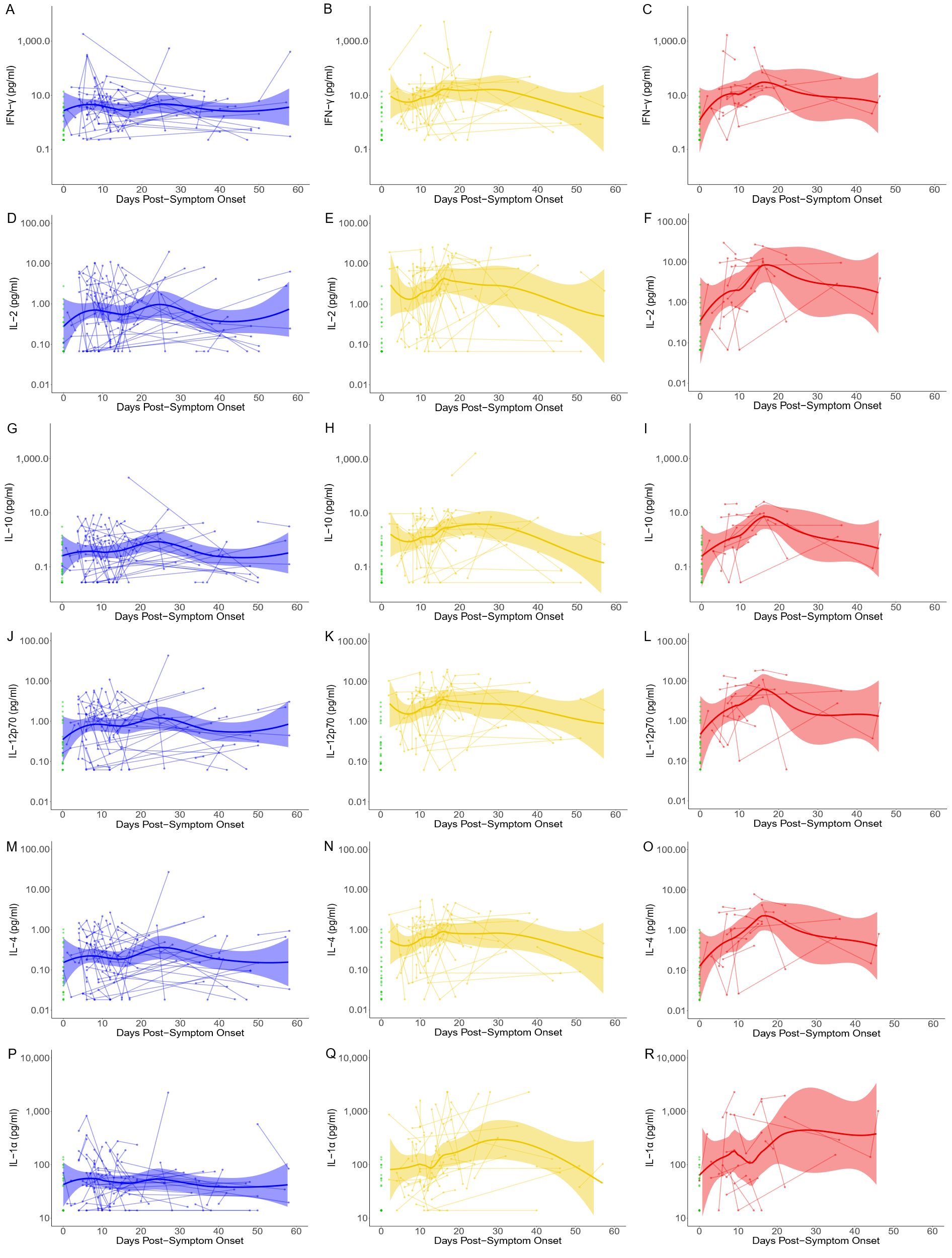


**Supplementary Figure 3 – Longitudinal sampling reveals distinct immune mediator kinetics between COVID-19 peak severity groups**

Immune mediator levels in sequential nasosorption samples from COVID-19 patients (n=153 patients, n=348 samples) were related to the duration of symptoms at the time of each sample collection. A-C) IFN-γ levels in Moderate (Blue), Severe (Yellow), and Fatal (Red) peak severity groups, respectively. D-F) IL-2 levels in Moderate, Severe, and Fatal peak severity groups, respectively. G-I) IL-10 levels in Moderate, Severe, and Fatal peak severity groups, respectively. J-L) IL-12p70 levels in Moderate, Severe, and Fatal peak severity groups, respectively. M-O) IL-4 levels in Moderate, Severe, and Fatal peak severity groups, respectively. P-R) IL-1α levels in Moderate, Severe, and Fatal peak severity groups, respectively. Healthy control samples are denoted on each panel as green circles at 0 Days Post-Symptom Onset. Individual patient samples are denoted by circles with connecting straight lines. Dynamic lines denote smoothened LOESS curves with shaded areas representing 95% confidence intervals.


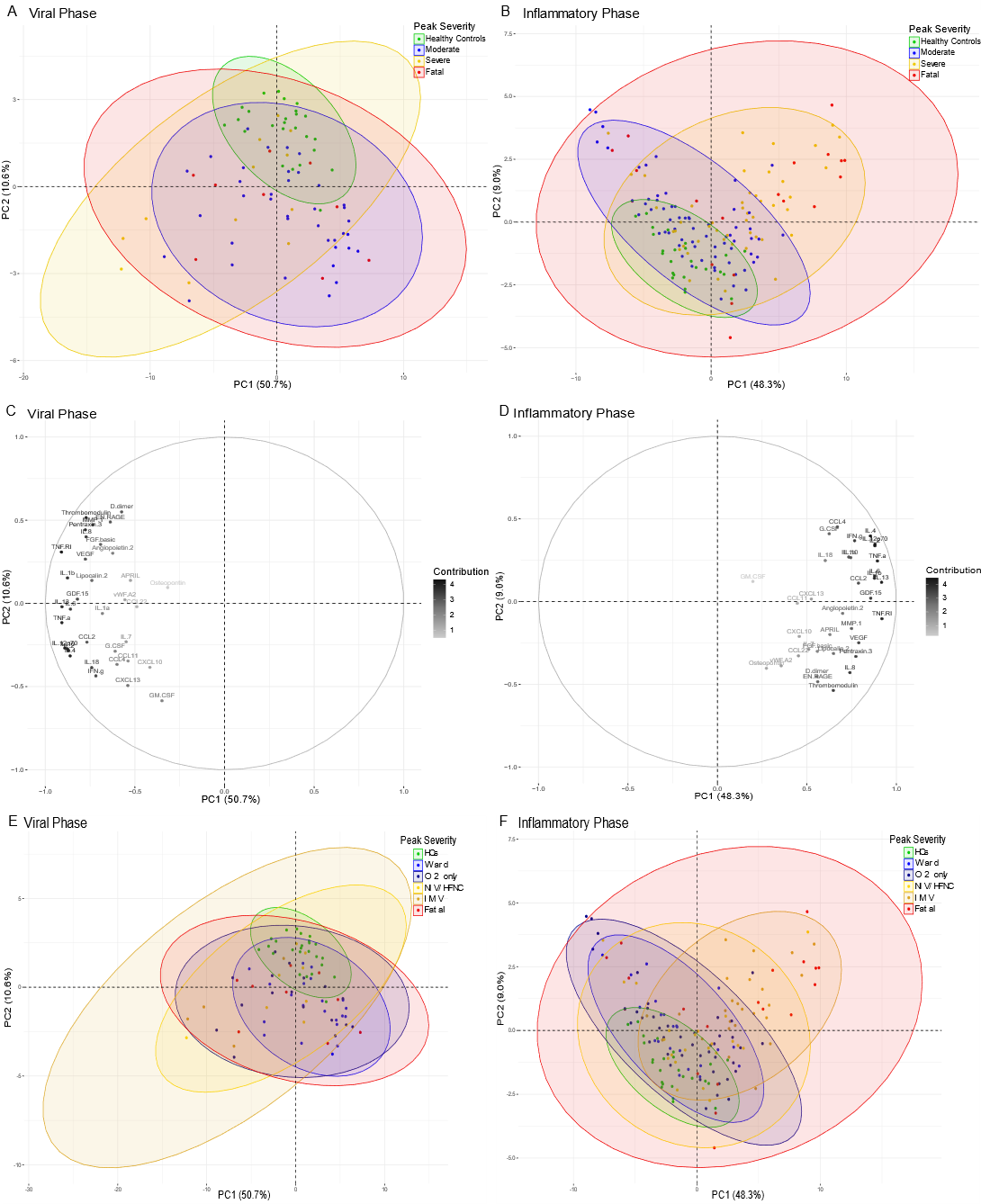


**Supplementary Figure 4 – Differences in mediators contributing to variation observed across PCs in viral and inflammatory phases**

Principal component analysis (PCA) of nasal mediator data within A) the Viral phase (0-5 days post-symptom onset, n=59) and B) the Inflammatory phase (6-20 days post-symptom onset, n=131). The relative contribution of each nasal mediator to the PCA variance observed amongst patients hospitalised with COVID-19 in C) the Viral phase and D) the Inflammatory Phase”. Panels A and B are annotated by peak disease severity (Blue=Moderate, Yellow=Severe, Red=Fatal).


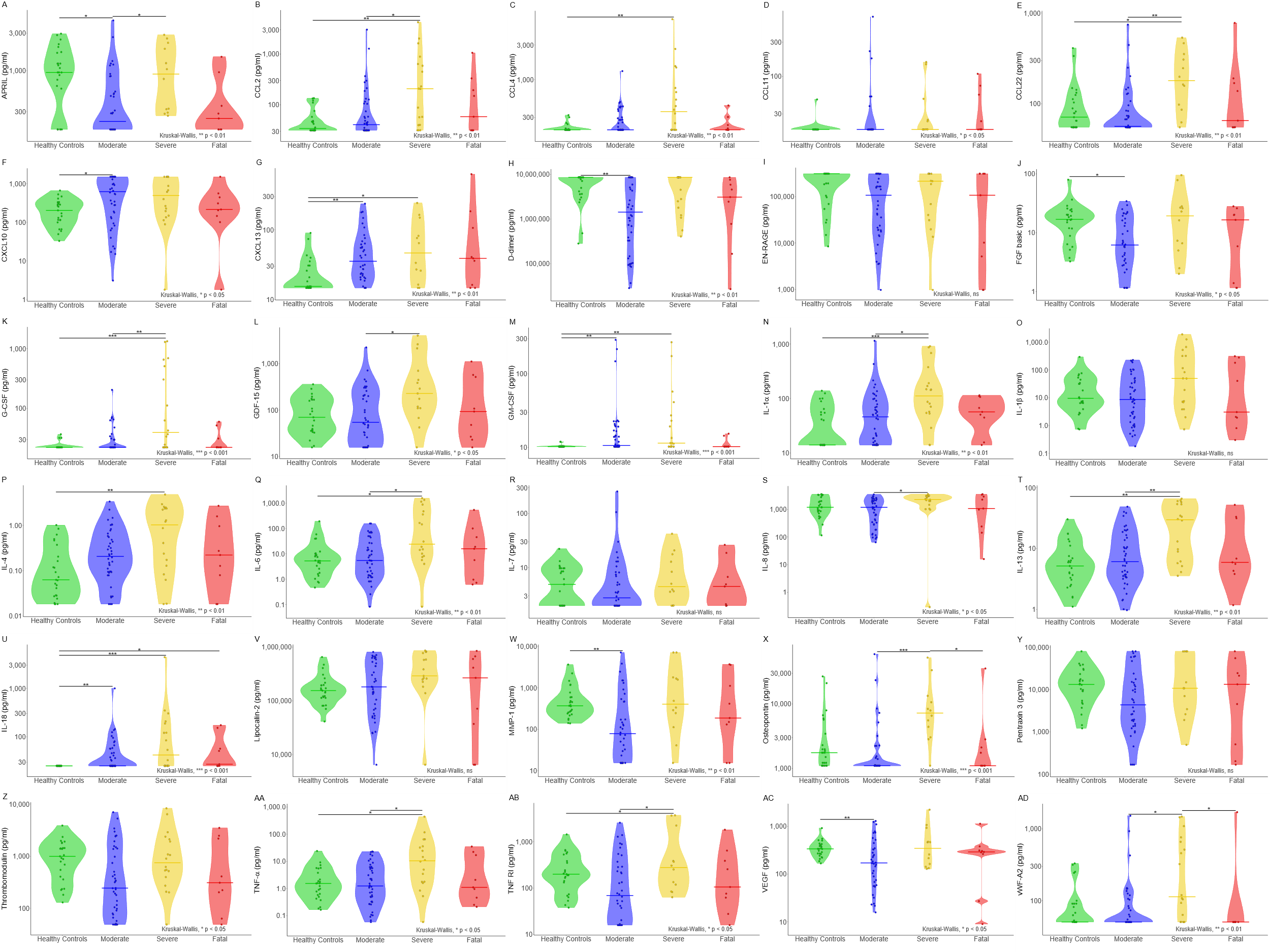


**Supplementary Figure 5 – Increased nasal mediator levels in the viral phase of the Severe peak severity group**

Individual mediator levels in nasosorption samples collected from patients during hospitalisation with COVID-19, displayed for healthy controls and peak COVID-19 severity groups during the Viral phase (0-5 days post-symptom onset). Immune mediators are ordered alphabetically. Statistical significance was tested using Kruskal-Wallis tests with Dunn’s p-value correction. *P<0.05, **P<0.01, ***P<0.001.


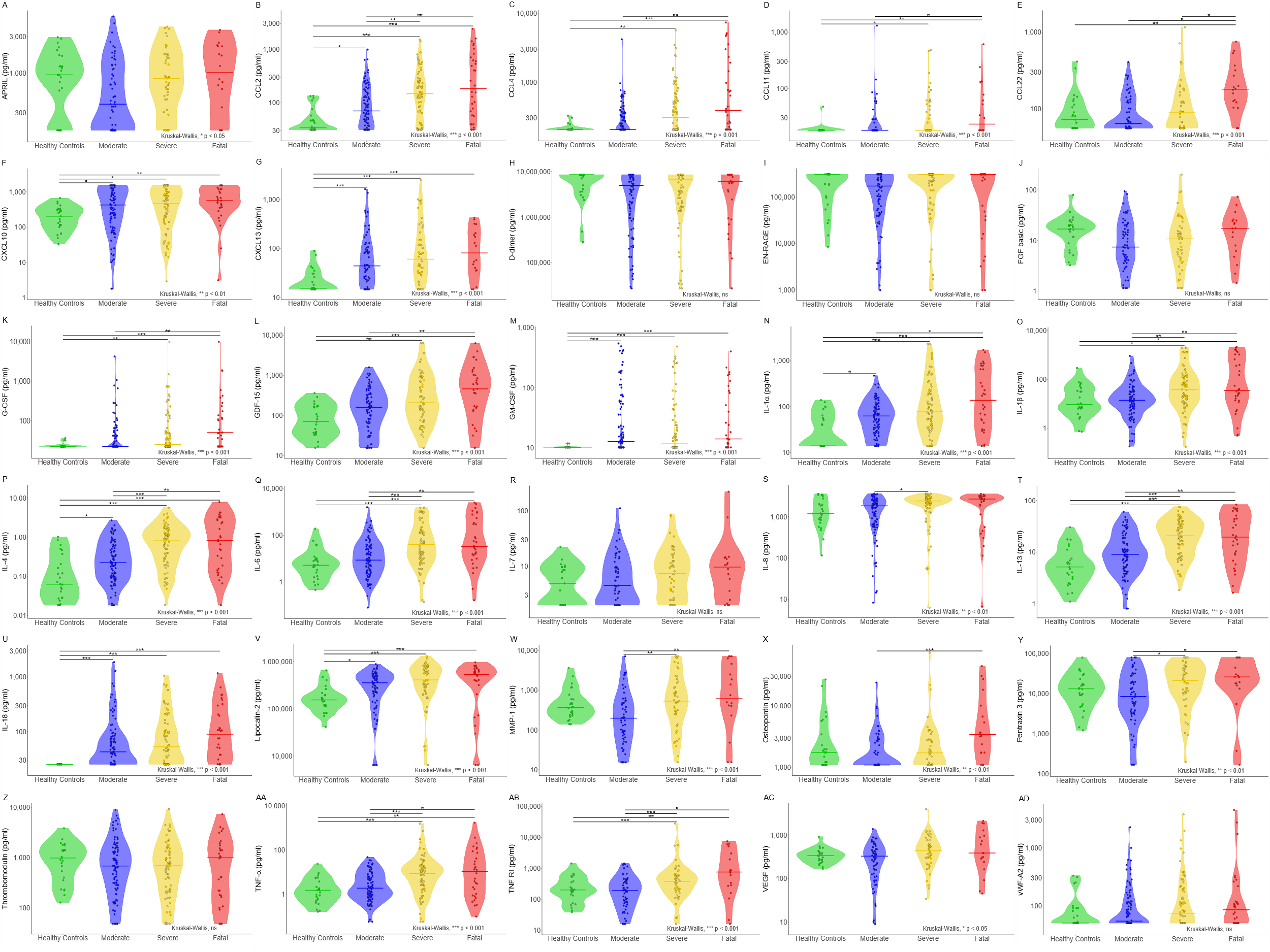


**Supplementary Figure 6 – Increased nasal mediator levels in the inflammatory phase of the Severe and Fatal peak severity groups**

Individual mediator levels in nasosorption samples collected from patients during hospitalisation with COVID-19, displayed for healthy controls and peak COVID-19 severity groups during the inflammatory phase (6-20 days post-symptom onset). Immune mediators are ordered alphabetically. Statistical significance was tested using Kruskal-Wallis tests with Dunn’s p-value correction. *P<0.05, **P<0.01, ***P<0.001.


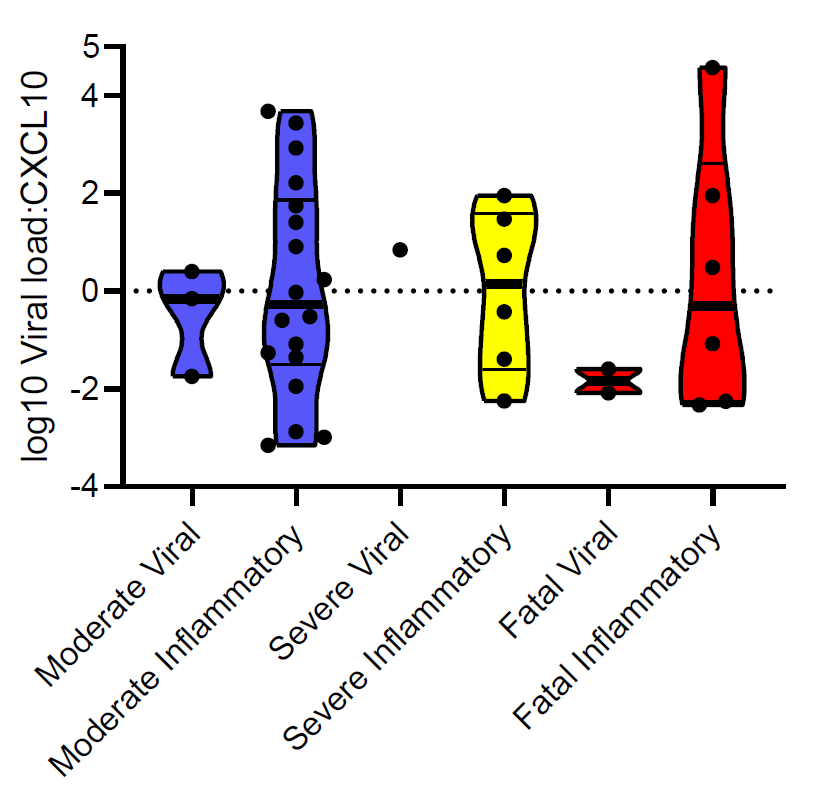


**Supplementary Figure 7 –SARS-CoV-2 viral load : CXCL10 levels between peak severity groups**

Nasal swab SARS-CoV-2 viral loads were related to CXCL10 levels in contemporaneous nasosorption samples collected from patients during hospitalisation with COVID-19 (n=36). Participants were grouped by peak COVID-19 severity during the Viral (0-5 days post-symptom onset) and Inflammatory phases (6-20 days post-symptom onset). All values are log10 transformed, with low values reflecting low CXCL10 levels for a given viral load. Thick lines denote group medians and thin lines denote quartiles. Statistical significance was tested using Kruskal-Wallis tests with Dunn’s p-value correction within either Viral or Inflammatory phases, yielding no significant differences.


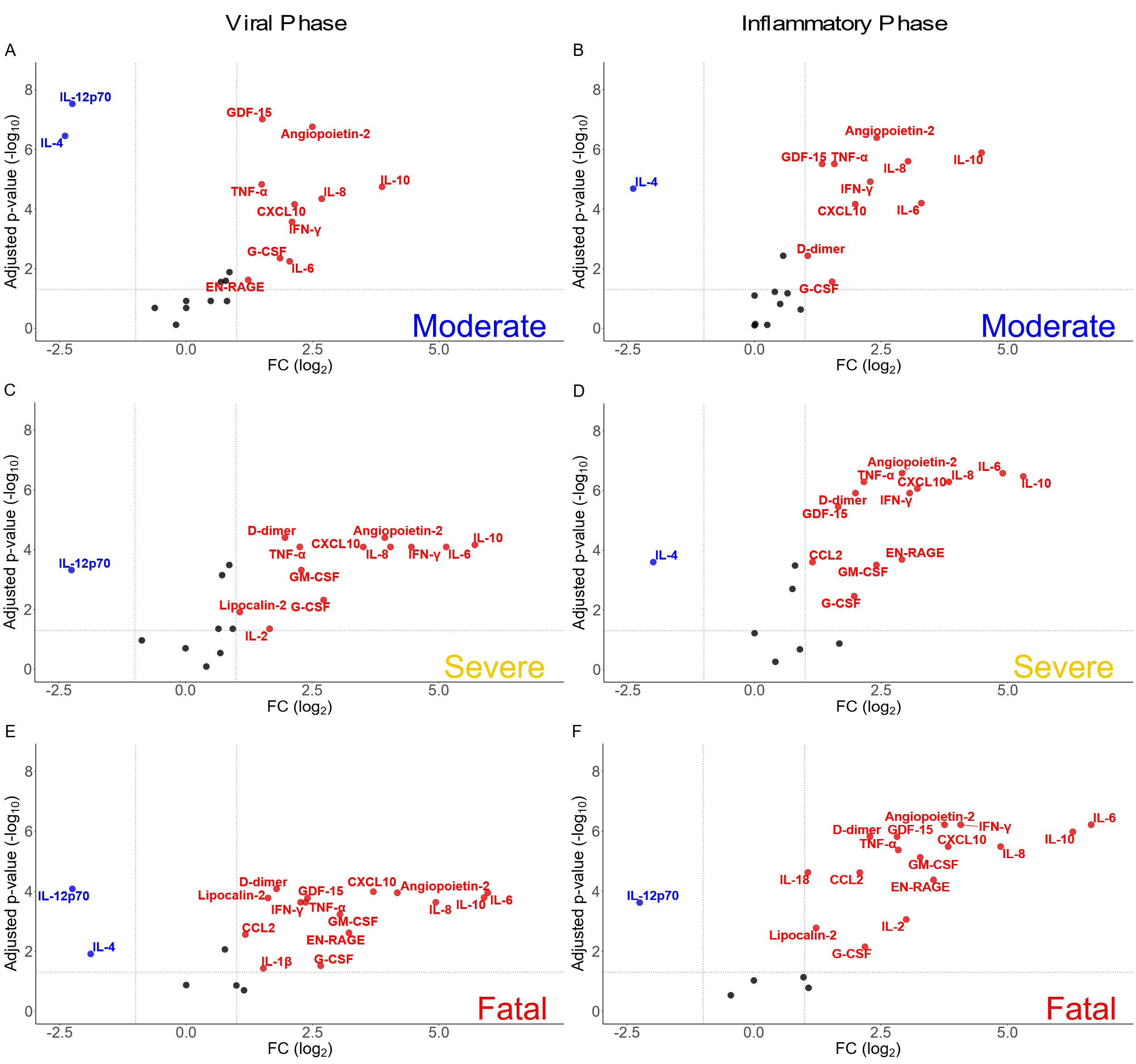


**Supplementary Figure 8 – Mediator levels in the plasma during the Viral and Inflammatory phases**

Mediator levels in the plasma of patients with COVID-19, grouped according to peak disease severity; A-B) Moderate, C-D) Severe, E-F) Fatal in the Viral (A, C, E; 0-5 symptom days) and Inflammatory (B, D, F; 6-20 symptom days) phases. Volcano plots display false-discovery rate adjusted Wilcoxon Rank-Sum *P*-values (cut-off 0.05) against log2 fold changes (cut-offs ≤-2 and ≥2) relative to levels in healthy control plasma.

**Supplementary Tables**

| **Nasal Mucosa** | **Healthy Controls** | **Moderate (WHO Scores 4-5)** | **Severe (WHO Scores 6-9)** | **Fatal (WHO Score 10)** |
| --- | --- | --- | --- | --- |
| **Demographics** | **Median (Range, Participant Counts)** | | | |
| Age | 35 (19-57, 25) | 61 (3-103, 142) | 57 (9-83, 92) | 67.5 (37-94, 40) |
| Age (Female) | 32 (19-56, 13) | 59 (3-95, 61) | 59 (9-71, 25) | 67.5 (37-88, 8) |
| Age (Male) | 38 (24-57, 12) | 61 (10-103, 80) | 56.5 (15-83, 66) | 67.5 (40-94, 32) |
| Age (Sex not specified) | - | 65 (65-65, 1) | 75 (75-75, 1) | - |
| Days Post-Symptom Onset | - | 8 (0-20, 142) | 11 (1-20, 92) | 9 (0-20, 40) |
| Days Post-Symptom Onset (Female) | - | 8 (0-20, 61) | 11 (2-20, 25) | 7.5 (3-16, 8) |
| Days Post-Symptom Onset (Male) | - | 8 (0-20, 80) | 11 (1-20, 66) | 10 (0-20, 32) |
| Days Post-Symptom Onset (Sex not specified) | - | 11 (11-11, 1) | 3 (3-3, 1) | - |
| Hospital Stay | - | 6 (0-67, 142) | 13.5 (0-94, 92) | 13 (3-71, 40) |
| Hospital Stay (Female) | - | 5 (0-51, 61) | 14 (0-94, 25) | 11 (7-17, 8) |
| Hospital Stay (Male) | - | 7 (0-67, 80) | 13 (0-84, 66) | 15 (3-71, 32) |
| Hospital Stay (Sex not specified) | - | 9 (9-9, 1) | 17 (17-17, 1) | - |
| Age (Sample Severity) | - | 63 (9-103, 163) | 56 (9-78, 67) | 70 (70-70, 1) |
| Days Post-Symptom Onset (Sample Severity) | - | 8 (0-20, 163) | 11 (2-20, 67) | 8 (8-8, 1) |
| Hospital Stay (Sample Severity) | - | 8 (0-71, 163) | 13 (0-94, 67) | 3 (3-3, 1) |
|  | | | | |
| **Measurements** | **Median (Range, Participant Counts)** | | | |
| Lymphocytes (10^9^/L) | - | 1 (0.2-3.2, 142) | 0.91 (0.2-7.3, 91) | 0.7 (0.2-2.17, 40) |
| Neutrophils (10^9^/L) | - | 5.985 (0.02-23.4, 142) | 6.66 (0.02-31.68, 91) | 7.935 (1.78-31.68, 40) |
| White Blood Cells/WBC (10^9^/L) | - | 7.95 (1.3-89, 142) | 8.635 (0.3-33.9, 91) | 10.25 (3.8-26.9, 40) |
| Platelets (10^9^/L) | - | 234 (21-964, 142) | 270.5 (21-817, 91) | 253 (21-586, 40) |
| Haematocrit (L/L) | - | 13.74 (0.3-51, 22) | 0.38 (0.19-44, 30) | 28 (0.25-39, 14) |
| C-reactive Protein/CRP (mg/L) | - | 69 (2-352, 142) | 102 (0.2-391, 91) | 163.6 (6-434.5, 40) |
| Procalcitonin/PCT (ng/ml) | - | - | 0.605 (0.37-3.34, 3) | 0.09 (0.09-0.09, 1) |
| Temperature (Celcius) | - | 37 (35.3-39.6, 34) | 36.7 (36.4-37.5, 13) | 37.4 (36.1-39.3, 12) |
| Heart Rate (bpm) | - | 87 (38-162, 35) | 79.5 (64-127, 11) | 88 (66-135, 9) |
| Systolic Blood Pressure (mmHg) | - | 125 (80-174, 45) | 110 (50-173, 38) | 107 (90-212, 16) |
| Respiratory Rate (breaths per min) | - | 20 (16-48, 33) | 24 (14-50, 11) | 20 (12-32, 9) |
| Fraction of Inspired Oxygen/FiO2 | - | 0.24 (0.21-0.6, 23) | 0.5 (0.21-1, 37) | 0.7 (0.21-1, 22) |
| Arterial Oxygen Saturation/SaO2 (%) | - | 94 (85-100, 61) | 92 (85-100, 41) | 89 (66.4-99.2, 29) |
| Lactate Dehydrogenase/LDH (units/L) | - | 364 (197-531, 2) | 397 (47-1485, 12) | 404.5 (242-674, 6) |
| Creatinine (μmol/L) | - | 76 (22-634, 45) | 74 (27-544, 41) | 75 (30-543, 24) |
| Creatinine Phosphokinase/CPK (mcg/L) | - | - | 208 (31-2377, 14) | 230 (37-1248, 5) |
| Bilirubin (μmol/L) | - | 8 (3-52, 28) | 9 (3-32, 30) | 10.5 (3-30, 18) |
| Glasgow Coma Scale/GCS (score) | - | 15 (15-15, 78) | 15 (3-15, 46) | 6 (3-15, 28) |
|  | | | | |
| **Disease Status** | **Participant Counts** | | | |
| Comorbidity | - | 21 (YES), 66 (NO) | 7 (YES), 47 (NO) | 9 (YES), 22 (NO) |
| Diabetes | - | 2 (YES), 85 (NO) | 0 (YES), 54 (NO) | 0 (YES), 31 (NO) |
| Obesity | - | 6 (YES), 73 (NO) | 10 (YES), 41 (NO) | 8 (YES), 22 (NO) |
| Cardiac | - | 17 (YES), 66 (NO) | 11 (YES), 42 (NO) | 11 (YES), 18 (NO) |
| Hypertension | - | 15 (YES), 21 (NO) | 6 (YES), 7 (NO) | 4 (YES), 7 (NO) |
| Pulmonary | - | 11 (YES), 72 (NO) | 1 (YES), 50 (NO) | 4 (YES), 25 (NO) |
| Asthma | - | 16 (YES), 67 (NO) | 6 (YES), 46 (NO) | 2 (YES), 27 (NO) |
| Renal | - | 9 (YES), 74 (NO) | 3 (YES), 49 (NO) | 4 (YES), 25 (NO) |
| Liver | - | 1 (YES), 35 (NO) | 0 (YES), 10 (NO) | 0 (YES), 9 (NO) |
| Dementia | - | 1 (YES), 34 (NO) | 0 (YES), 10 (NO) | 1 (YES), 8 (NO) |
| Cancer | - | 1 (YES), 34 (NO) | 0 (YES), 10 (NO) | 0 (YES), 9 (NO) |
| Haemotological | - | 0 (YES), 35 (NO) | 1 (YES), 9 (NO) | 2 (YES), 7 (NO) |
| AIDS/HIV | - | 0 (YES), 35 (NO) | 0 (YES), 10 (NO) | 0 (YES), 9 (NO) |

**Supplementary Table 1 – Patient and participant demographics**

| **Nasal Mucosa** | | **Age** | **Days Post-Symptom Onset** |
| --- | --- | --- | --- |
| **Peak Severity** | **Replicate** | **Median (Range, Participant Counts)** | |
| Moderate (WHO Scores 4-5) | 1 | 58 (28-95, 56) | 9 (0-50, 56) |
|  | 2 | 58 (28-95, 56) | 14 (2-58, 56) |
|  | 3 | 56 (29-89, 18) | 19 (12-84, 18) |
|  | 4 | 73 (65-81, 3) | 58 (32-89, 3) |
| Severe (WHO Scores 6-9) | 1 | 58 (24-83, 41) | 11 (2-51, 41) |
|  | 2 | 58 (24-83, 41) | 16 (4-57, 41) |
|  | 3 | 57 (44-83, 11) | 25 (10-51, 11) |
|  | 4 | 44 (44, 1) | 19 (19, 1) |
| Fatal (WHO Score 10) | 1 | 67 (37-88, 18) | 8 (0-22, 18) |
|  | 2 | 67 (37-88, 18) | 13 (2-44, 18) |
|  | 3 | 67 (56-74, 5) | 22 (10-46, 5) |

**Supplementary Table 2 – Characteristics of participants included in the analysis of longitudinal nasal samples**

|  | | **Healthy Controls** | **Moderate (WHO Scores 4-5)** | **Severe (WHO Scores 6-9)** | **Fatal (WHO Score 10)** |
| --- | --- | --- | --- | --- | --- |
| **Compartment** | **Demographics** | **Median (Range, Participant Counts)** | | | |
| Nasal Mucosa | Age | 35 (19-57, 25) | - | - | - |
| Plasma | Age | 56 (45-71, 15) | - | - | - |
| Nasal Viral Phase | Age | - | 63 (3-103, 44) | 56 (15-76, 18) | 82 (37-94, 9) |
|  | Days Post-Symptom Onset | - | 4 (0-5, 44) | 4 (1-5, 18) | 3 (0-5, 9) |
| Nasal Inflammatory Phase | Age | - | 59 (10-95, 98) | 57 (9-83, 74) | 67 (41-84, 31) |
|  | Days Post-Symptom Onset | - | 10 (6-20, 98) | 13 (6-20, 74) | 11 (6-20, 31) |
| Plasma Viral Phase | Age | - | 55 (16-93, 69) | 55 (9-93, 19) | 74 (42-85, 14) |
|  | Days Post-Symptom Onset | - | 4 (1-5, 69) | 4 (1-5, 19) | 4 (1-5, 14) |
| Plasma Inflammatory Phase | Age | - | 57 (23-90, 141) | 58 (15-86, 131) | 67 (44-84, 42) |
|  | Days Post-Symptom Onset | - | 10 (6-20, 141) | 12 (6-20, 131) | 11 (6-19, 42) |

**Supplementary Table 3 – Patient and participant demographics for both nasal and plasma samples included in the analysis of Viral and Inflammatory phases**
