# Supplementary figures and images for: Delayed Mucosal Antiviral Responses Despite Robust Peripheral Inflammation in Fatal COVID-19
Source: J Infect Dis. 2023 Dec 22;230(1):e17–29. doi: 10.1093/infdis/jiad590 (PMC11272059; doi:10.1093/infdis/jiad590)

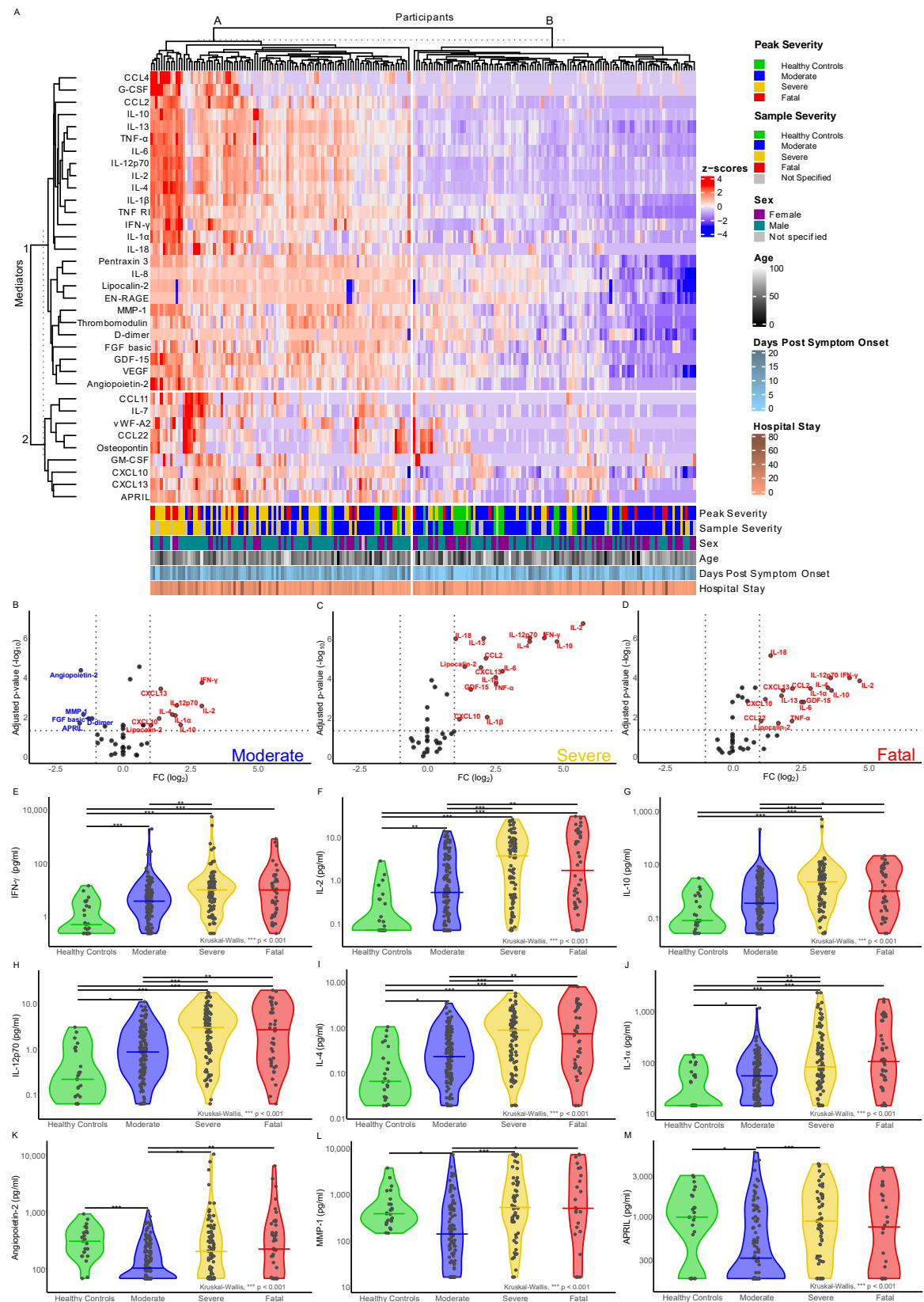

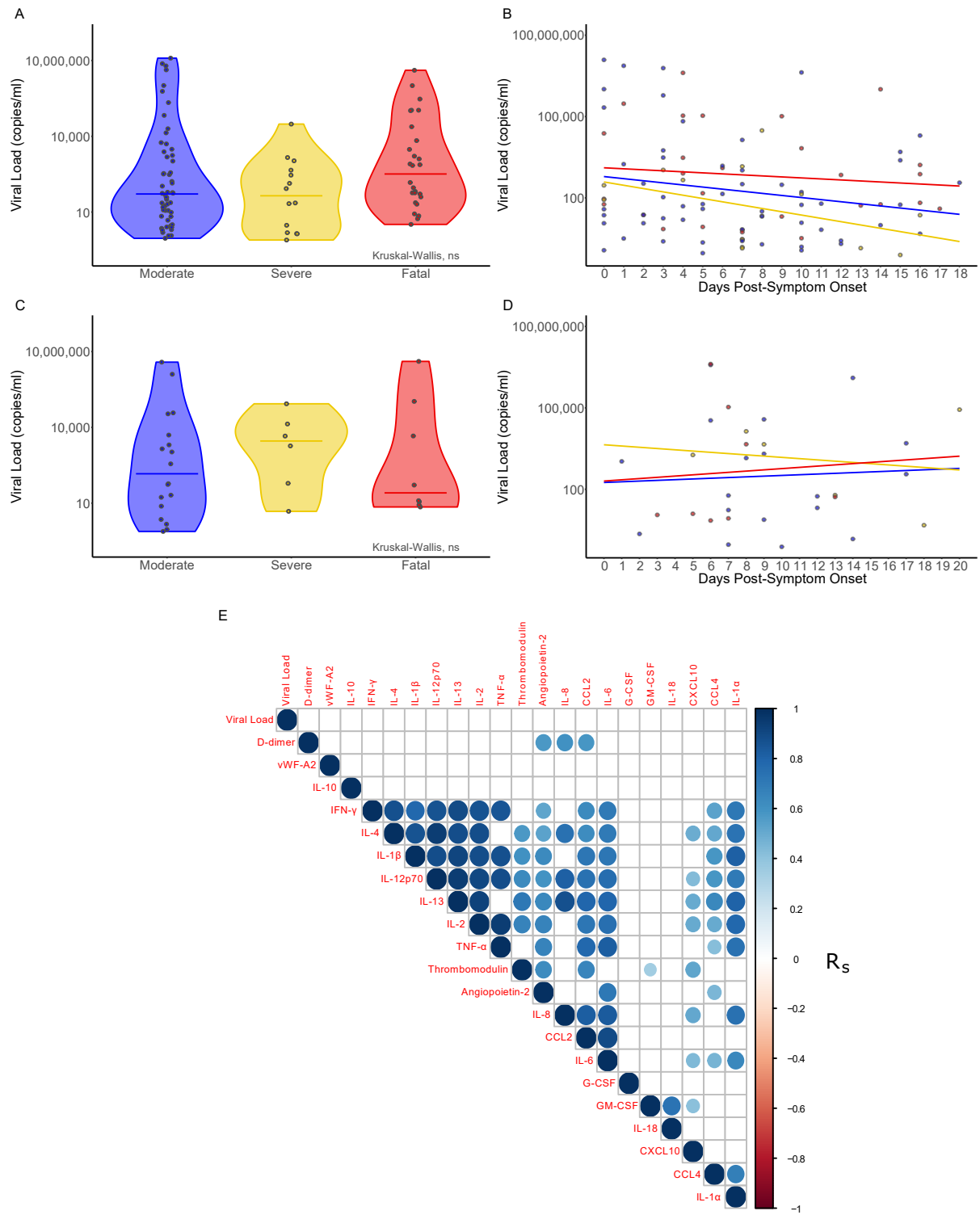

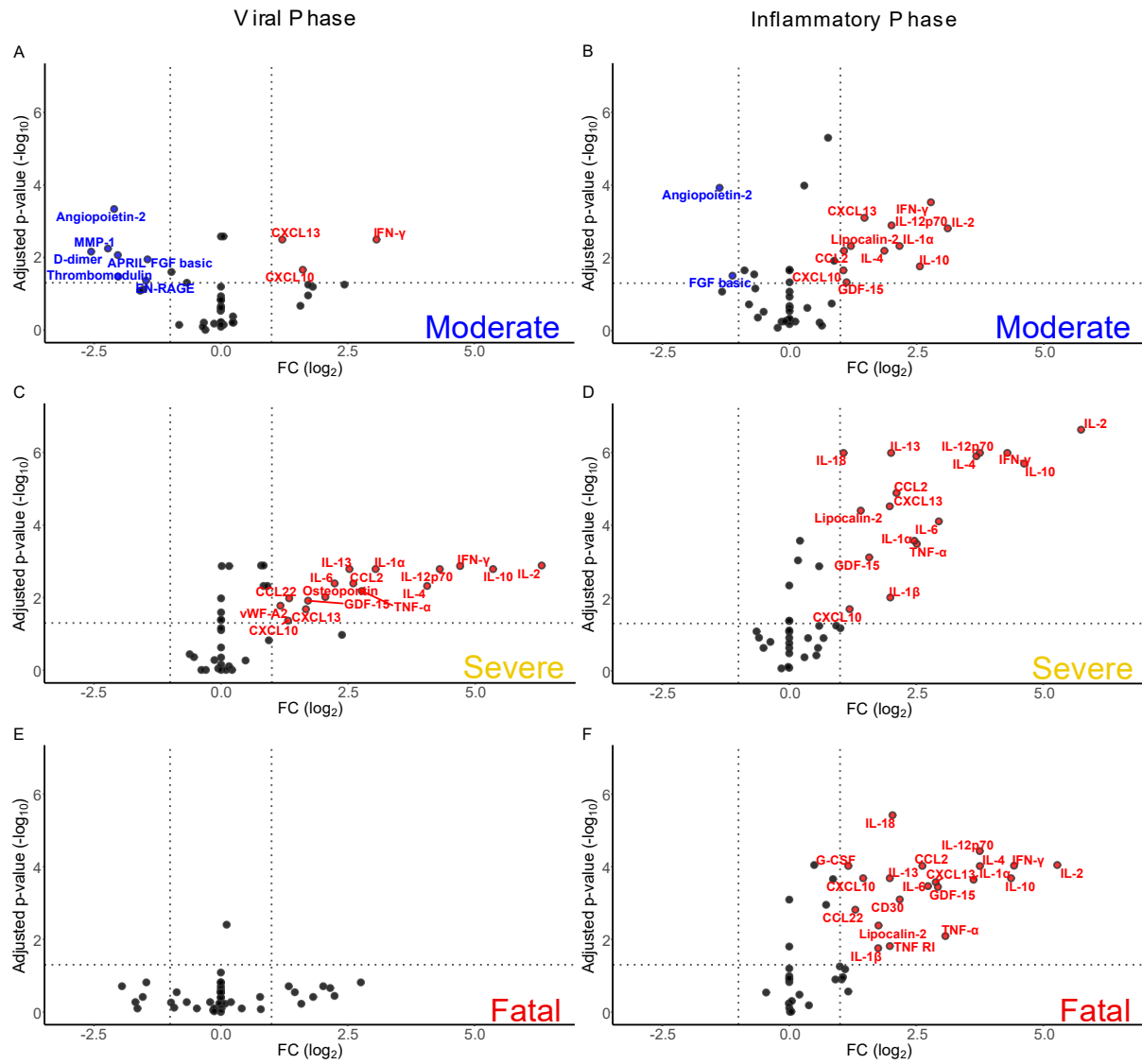

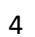

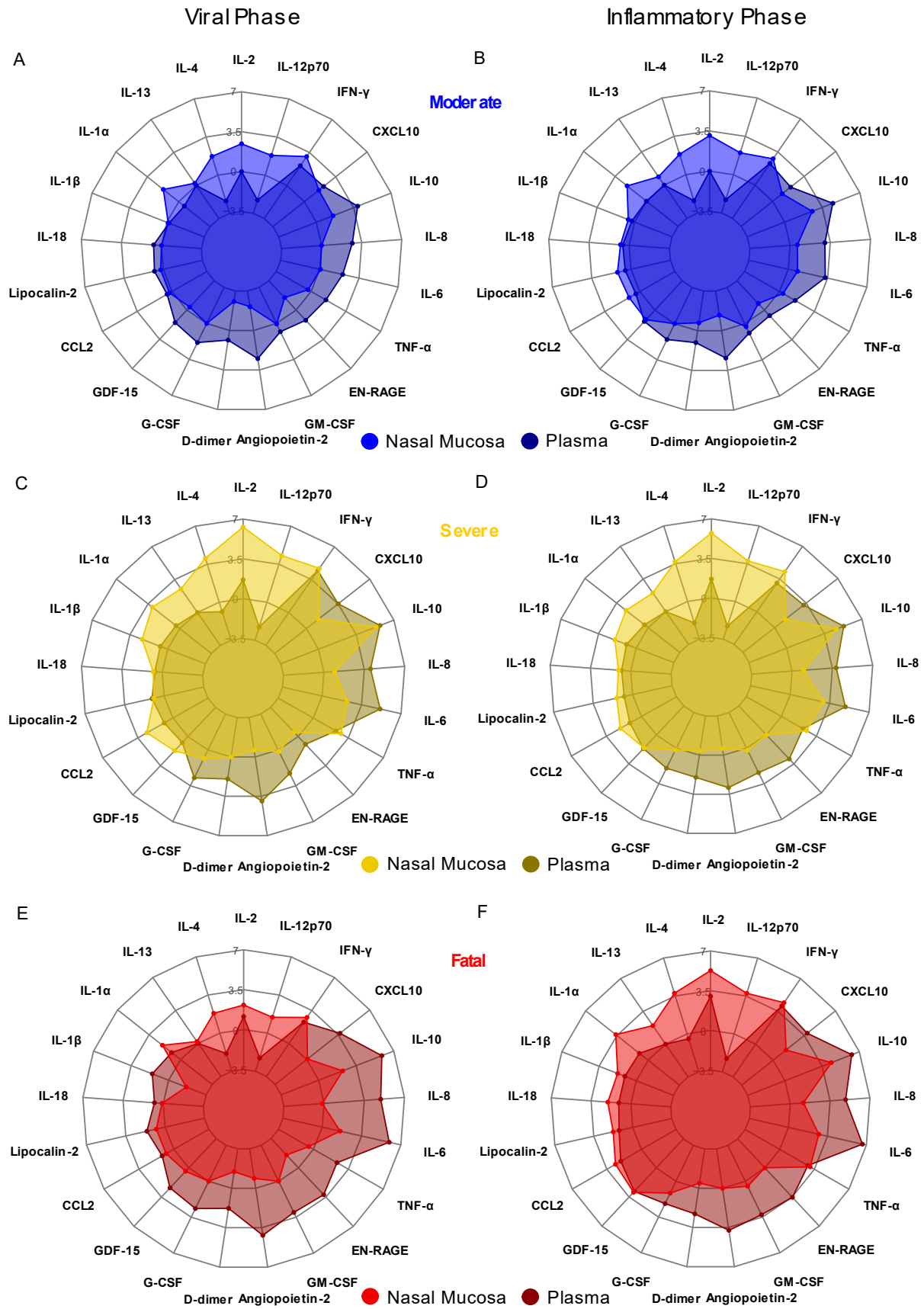

Supplement: jiad590_Supplementary_Data [file jiad590_supplementary_data.zip › JID ISARIC Mucosal Mediators_AllFigures_R1.pdf]
